# Genomic introgression mapping of field-derived multiple-anthelmintic resistance in *Teladorsagia circumcincta*

**DOI:** 10.1371/journal.pgen.1006857

**Published:** 2017-06-23

**Authors:** Young-Jun Choi, Stewart A. Bisset, Stephen R. Doyle, Kymberlie Hallsworth-Pepin, John Martin, Warwick N. Grant, Makedonka Mitreva

**Affiliations:** 1 McDonnell Genome Institute, Washington University School of Medicine, Saint Louis, Missouri, United States of America; 2 AgResearch, Hopkirk Research Institute, Palmerston North, New Zealand; 3 Department of Animal, Plant and Soil Sciences, La Trobe University, Melbourne, Victoria, Australia; 4 Department of Medicine, Washington University School of Medicine, Saint Louis, Missouri, United States of America; Northwestern University, UNITED STATES

## Abstract

Preventive chemotherapy has long been practiced against nematode parasites of livestock, leading to widespread drug resistance, and is increasingly being adopted for eradication of human parasitic nematodes even though it is similarly likely to lead to drug resistance. Given that the genetic architecture of resistance is poorly understood for any nematode, we have analyzed multidrug resistant *Teladorsagia circumcincta*, a major parasite of sheep, as a model for analysis of resistance selection. We introgressed a field-derived multiresistant genotype into a partially inbred susceptible genetic background (through repeated backcrossing and drug selection) and performed genome-wide scans in the backcross progeny and drug-selected F2 populations to identify the major genes responsible for the multidrug resistance. We identified variation linking candidate resistance genes to each drug class. Putative mechanisms included target site polymorphism, changes in likely regulatory regions and copy number variation in efflux transporters. This work elucidates the genetic architecture of multiple anthelmintic resistance in a parasitic nematode for the first time and establishes a framework for future studies of anthelmintic resistance in nematode parasites of humans.

## Author summary

*Teladorsagia circumcincta* is an economically significant nematode (roundworm) pathogen affecting sheep and goats in temperate regions of the world. The widespread use of prophylactic treatment has resulted in rapid selection for anthelmintic (anti-worm drug) resistance in this and other species of livestock parasites. The mechanism of resistance is not well understood because most studies have focused on the role of candidate genes using simplistic models of single gene selection, despite evidence that the evolution of resistance is more complex. Here, we report on a comprehensive whole-genome analysis that elucidated resistance-associated genes, which was facilitated by developing a pair of *T*. *circumcincta* strains sharing a largely common genetic background but differing markedly in their susceptibility to anthelmintic drugs. The results show that multiple genetic factors contribute to anthelmintic resistance in a variety of ways, including possible reduction/modulation in target site sensitivity, reduced target site expression, and increased drug efflux, to name a few. This suggests that drug resistance in these parasites is a multifactorial quantitative trait rather than a simple discrete Mendelian character. With this study, we established a genomics-based experimental paradigm for investigating anthelmintic resistance, at a time when its medical importance is rapidly increasing.

## Introduction

Anthelmintic resistance is already a global problem for agriculture and a growing concern in relation to human pathogens [1, 2]. In the absence of effective vaccines, treatment and prophylaxis of helminthiases rely on a limited number of chemotherapeutic agents whose efficacy is increasingly undermined by the selection and spread of resistant parasites. Although fundamental to our ability to conserve sensitivity to existing drugs and to design improved interventions, the molecular and population genetic bases of anthelmintic resistance remain inadequately understood [3, 4]. To date, most studies have focused on the role of individual candidate genes such as drug targets or transporters. However, while such studies have been instrumental in identifying some causal genetic variants associated with drug resistance, frequently they have accounted for only a proportion of the drug resistant phenotypes present in the population, suggesting that the trait probably has a complex multi-genic nature [5–7]. Efforts to comprehensively map functional polymorphisms and to clarify the genotype-phenotype relationships in anthelmintic resistance have been challenging, given the relatively poor genetic and experimental tractability of helminth systems, which has impeded genome-wide studies beyond targeted analysis of particular candidate genes [8].

The problem of anthelmintic resistance is most severe in the trichostrongylid nematodes of livestock and particularly those infecting small ruminants such as sheep and goats [2]. The troubling propensity of these parasites to develop drug resistance has been attributed to their enormous effective population size and the resulting genetic diversity upon which selection is able to act [9, 10] and although variation is a prerequisite for selection, the extreme genetic heterogeneity in parasite populations often confounds the identification or association of genetic components contributing toward anthelmintic resistance. This difficulty is further hampered by factors such as the degree of parasite population connectivity due to parasite and/or host movement [11], the influence of population size and life history traits on genetic drift within parasite subpopulations, and the variation in local parasite management strategies, all of which likely influence the ability to detect and correctly interpret genetic differentiation between anthelmintic resistant and susceptible parasites [10]. Our approach towards identifying drug resistance associated genes involved the controlled crossing of a multidrug-resistant parasite strain with a characterized susceptible strain followed by repeated backcrossing and drug selection, which resulted in the introgression of resistance associated alleles into a largely susceptible, partially inbred genetic background. By identifying the alleles derived from the original resistant parent in the resulting backcrossed progeny, it was possible to generate a genetic map of resistance loci within the genome. Similar approaches have been used in mapping drug resistance associated loci in *Haemonchus contortus* [12, 13] and elucidating the genetic basis of drug resistance in some trematode parasite species [14].

In this study, we extended our previous work [15] by combining a genetic introgression approach with whole-genome sequencing to further elucidate the genetic basis of field-derived multiple-anthelmintic resistance in *Teladorsagia circumcincta*, the most economically important nematode pathogen affecting sheep and goats in temperate regions of the world. *T*. *circumcincta* is a monoxenous, obligately sexual species that infects the fourth stomach (abomasum) of small ruminants, leading to reduced wool, milk and meat production, and in severe cases, death. Widespread anthelmintic resistance has arisen in this trichostrongylid parasite, including multiple-anthelmintic resistance to all major broad-spectrum drug classes available prior to 2008 (i.e., benzimidazoles, imidazothiazoles and macrocyclic lactones, which target microtubule polymerization, nicotinic acetylcholine receptors and glutamate-gated chloride channels respectively) [16] and also to the more recently released amino-acetonitrile derivatives [17]. Through controlled genetic crosses set up by surgical transplantation, we undertook a serial backcrossing experiment that aimed to introgress the resistance-related genes from a field isolate into the genomic background of a partially inbred susceptible recurrent parental strain. Using this partially inbred susceptible parental strain, we generated a draft reference genome of *T*. *circumcincta* by *de novo* assembly, which was subsequently used to conduct comparative genome-wide single nucleotide and copy number variant analyses of the resistant strain. In addition, using a combination of pooled (Pool-seq) and individual (ddRAD-seq) genome sequencing and RNA-seq, we identified genes with differential patterns of diversity associated with multiple-anthelmintic resistance.

## Results and discussion

### The reference genome sequence of *T*. *circumcincta*

**Fig 1:**
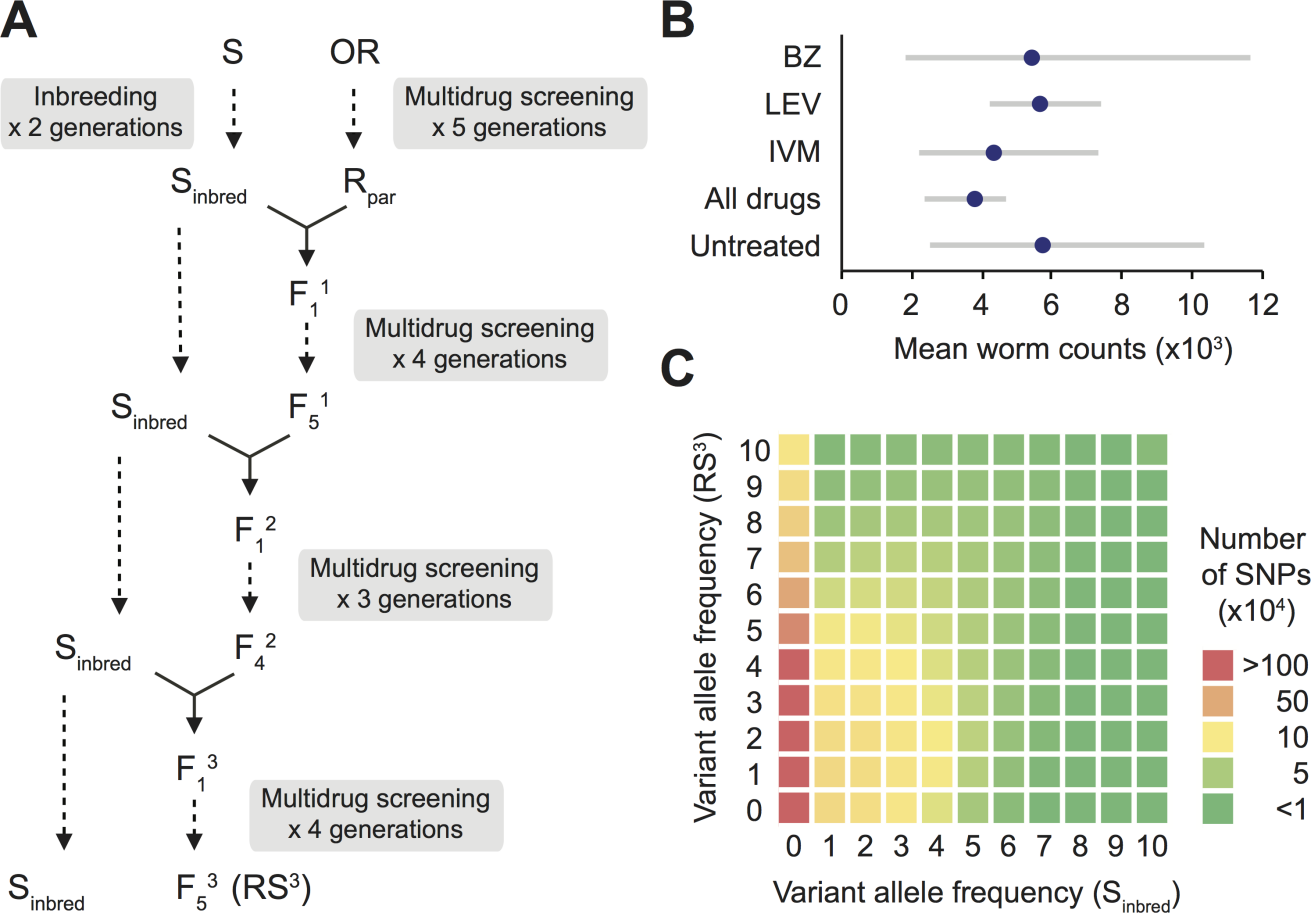
Generation of mapping populations in *Teladorsagia circumcincta*. (A) Introgressing field-derived multiple-anthelmintic resistance alleles into an inbred anthelmintic susceptible genetic background. Oxfendazole (BZ), levamisole (LEV) and ivermectin (IVM) were used for multidrug screening. (B) Efficacies of BZ, LEV and IVM against the multiple-anthelmintic resistant R_par_ strain of *T*. *circumcincta* in goat kids. Back-transformed square-root mean worm counts were presented with range of actual counts. No statistically significant worm count differences were detected between any of the groups (Wilcoxon rank-sum test, n = 6 for each treatment group). (C) Two-dimensional variant allele frequency spectra for S_inbred_ and RS^3^ *T*. *circumcincta* populations. Both horizontal and vertical axes are in units of 10%. Bi-allelic SNPs were used after subsampling to a uniform coverage of 10× for both strains because differences in mean sequencing depths could lead to a systematic bias in polymorphism detection sensitivity.

We generated a draft genome of *T*. *circumcincta* using the partially inbred anthelmintic susceptible strain (S_inbred_) which was used as the recurrent parent in the backcrossing program undertaken to introgress anthelmintic resistance-associated genes/alleles into a susceptible genetic background (Fig 1A and 1B). The draft nuclear genome of ~701 Mb (93.4% CEGMA completeness [18]) comprises 81,730 supercontigs (S1 Table), with 35.0% (28,621) of the supercontigs accounting for 90% of the genome. The GC content was 44.8%. The amino acid composition was comparable to that of other phylogenetically close parasitic species such as *Necator americanus* or non-parasitic *Caenorhabditis elegans* (S2 Table). In total, 1,583 repeat families were predicted and annotated, spanning 38.5% of the genome (S3 Table). We predicted a total of 25,532 protein-encoding genes, representing 2.3% of the genome at an average density of 36.4 genes per Mb with an average GC content of 47.8%. Compared to *C*. *elegans*, the gene density in *T*. *circumcincta* is lower and the average size of gene loci is larger (Mann–Whitney *U* test, *P* < 2.2 × 10^−16^) with longer introns (S1 Table). The majority of predicted genes (80.6%) were supported by transcriptional evidence from mixed-sex adult worm samples with RNA-seq coverage of at least 50% of the length of the annotated coding exons. We predicted secreted proteins (1,603 classical and 9,642 non-classical secretion) and putative membrane-bound proteins (3,749), representing 44% and 15% of the proteome respectively. Functional annotation of deduced proteins on the basis of primary sequence similarity comparisons identified 4,456 unique InterPro domains, 1,563 Gene Ontology terms, and 7,458 KEGG Orthology groups, for 66%, 51%, and 64% of the *T*. *circumcincta* genes respectively. When considered together, 78% of all *T*. *circumcincta* genes had some form of putative functional annotation. In spite of our inbreeding (two generations of sibling mating) efforts to reduce heterozygosity in preparation for genome sequencing, the quality of the final assembly still suffered from residual heterozygosity, which is consistent with previously reported genome assemblies of obligate outcrossing nematode species with a large effective population size [19, 20]. Although polymorphic haplotypes can be collapsed into consensus sequences during assembly, high genetic diversity tends to result in a fragmented, larger-than-expected assembly [21]. Reliably discriminating uncollapsed alleles from truly paralogous loci remains a significant challenge, and this caveat calls for a careful interpretation of our reference-alignment-based variant analysis.

### Introgression mapping of multiple-anthelmintic resistance loci

To analyze genetic variation between the RS^3^ and S_inbred_ strains, whole genome re-sequencing analysis was conducted using DNA obtained from pools of 300–500 mixed-sex worms. Approximately 92-fold coverage of the genome was obtained in total across the populations (43.6× and 48.3× from RS^3^ and S_inbred_ populations, respectively). Based on the depth of coverage, mapping quality, and gapped regions across all loci, 68.8% of the genome (482.3/700.6 Mb) and 90.6% of the coding sequences (14.9/16.4 Mb) were estimated to have at least the minimum sequence coverage for variant detection in both populations (S4 Table). A set of 17.6 million SNPs was obtained, of which 17.2 million (97.8%) were bi-allelic. The number of segregating (polymorphic) sites was overall ~2-fold lower for the S_inbred_ strain than for the RS^3^ strain (7,354,798 vs 16,489,377) (*see* S5 Table) indicating that the partial inbreeding strategy we adopted to reduce heterozygosity in the susceptible reference genome was successful. While a relatively small proportion of SNPs were differentially fixed in the two populations (S_inbred_: 4,094; RS^3^: 147,114 / 17,176,467), there was a notable excess of private SNPs in the RS^3^ population (9,617,901 + 19,932 cf 422,182 + 81,072) which were most likely introgressed from the resistant parent strain, R_par_. In addition to private R_par_ derived SNPs, the majority of SNPs observed in the S_inbred_ population were also segregating in the RS^3^ population (6,851,544 / 7,354,798) (S5 Table), as expected from the introgression strategy. Two-dimensional allele frequency spectra based on the bi-allelic sites illustrates this asymmetric distribution of private alleles with concentration of counts in cells along the vertical axis representing the RS^3^ population (Fig 1C). The observed pattern is consistent with the expected outcome of our experimental design which relied on a high level of genetic divergence between the two parental isolates (S_inbred_ and R_par_) and a unidirectional gene flow driven by the repeated use of S_inbred_ in backcrossing. In both populations, low-frequency SNPs were in deficit relative to neutral expectations (genome-wide Tajima’s D: 2.08 and 2.10 for S_inbred_ and RS^3^ respectively), likely due to a combination of ascertainment bias resulting from the limited sampling depth, the exclusion of singleton polymorphisms and the random loss of rare alleles following the inbreeding and introgression strategies employed in the construction of these strains. In addition, the observed level of genetic variability, particularly in the RS^3^ population, may be an underestimation considering a possible mapping bias against non-reference alleles. While these biases have the potential to increase uncertainty in population genetic parameter estimation, they likely have limited impact on our ability to detect outlier genomic regions showing the most extreme levels of divergence between the RS^3^ and the S_inbred_ populations.

**Fig 2:**
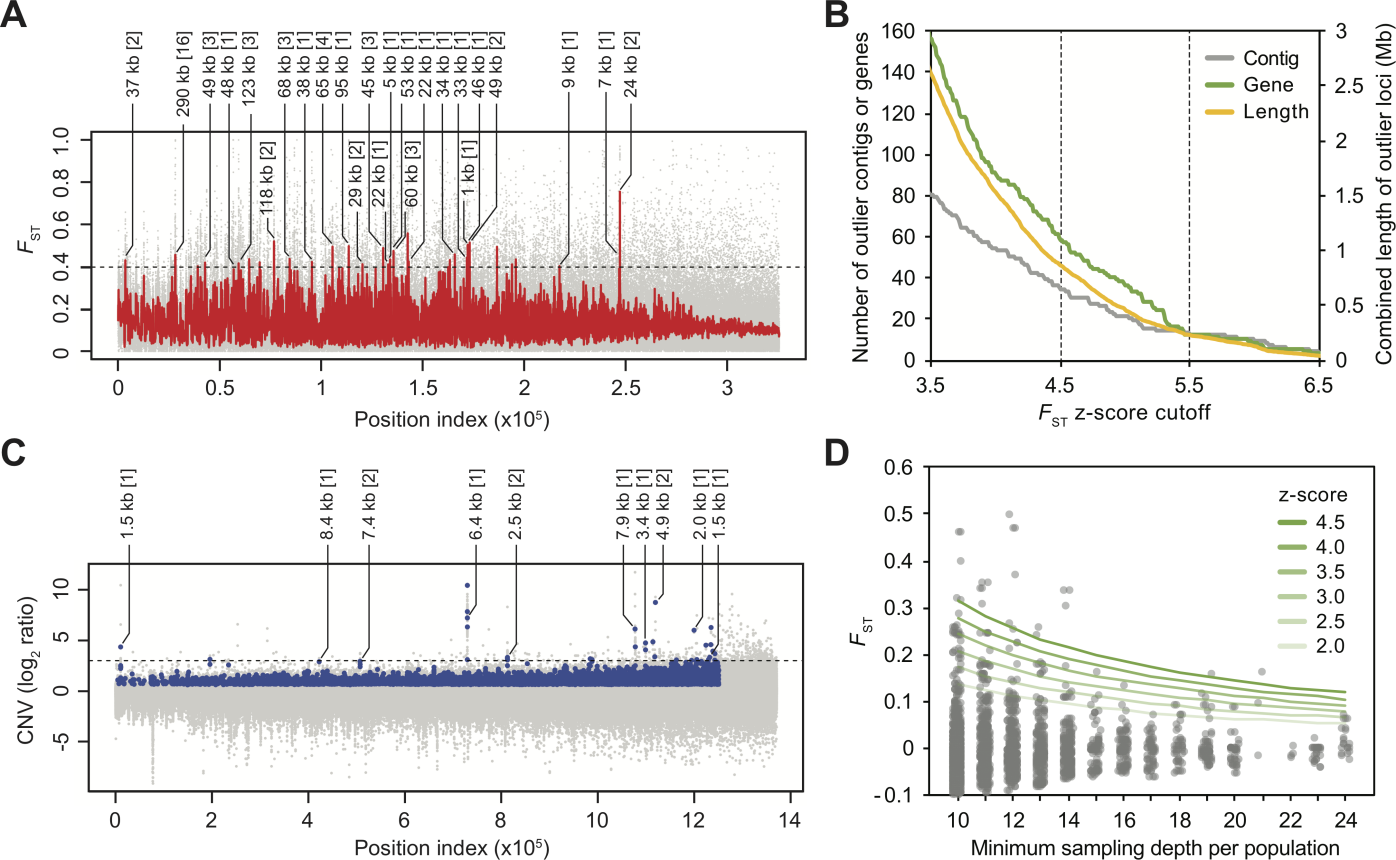
Genome-wide scan of fixation index (*F*_ST_) and copy number variation (CNV) between S_inbred_ and RS^3^ populations of *Teladorsagia circumcincta*. (A) Mean *F*_ST_ values for 1-kb sliding windows (grey) were subjected to kernel smoothing (red) to locate contiguous regions of the genome with high levels of population differentiation. Outlier regions (above the dashed line *F*_ST_ = 0.40 (z-score = 4.5)) were identified based on the empirical distribution of the smoothed *F*_ST_ values. The length and the number of genes per region (in brackets) are indicated for protein coding outlier loci. Due to the lack of information regarding the long-range relationship of the scaffold sequences, numerical index was used as the unit of relative location along the horizontal axis instead of the absolute genomic coordinates. Within each scaffold the order of windows followed the genomic coordinates. A total of ~325,000 windows were included in the analysis. (B) Total combined length of outlier regions, number of overlapping contigs and genes under different *F*_ST_ z-score cutoff values. (C) CNV was presented as the ratio of RS^3^:S_inbred_ normalized depth. Raw read count ratios (grey) and statistically significant CNV regions (blue). Top 10 outlier regions that contain protein-coding genes (log_2_ ratio >2.9; above dashed line) were identified (Table 2). The length and the number of genes per region (in brackets) were indicated. (D) *F*_ST_ of ddRAD-seq derived SNP markers between ivermectin-screened and drug-naïve F2 mapping populations of *T*. *circumcincta* (n = 24 for each population). Outlier loci were determined using z-score cutoff values based on the empirical distribution of *F*_ST_ estimates in each sampling depth category (represented as green lines of increasing intensity, ranging from a z-score of 2 to 4.5 in 0.5 increments).

In the RS^3^ parasites we expected the introgressed alleles associated with anthelmintic resistance to be contained in the divergent genomic regions originating from the R_par_ field isolate, which were maintained by drug selection in the face of repeated gene flow from the S_inbred_ reference strain. We further expected that independent meiotic recombination events would lead to variation in the introgression break points among the haplotypes segregating in the RS^3^ population such that, at the population level, a gradient of allelic divergence would be created peaking around the directly selected loci. A genome-wide scan of *F*_ST_ following kernel smoothing resulted in demarcation of contiguous regions of the genome with high levels of population differentiation, representing putative introgression blocks. Outlier regions were determined on the basis of the empirical distribution of the smoothed *F*_ST_ values (Fig 2A) with the goal of prioritizing candidate variants under anthelmintic selection as specific targets for future functional studies. Using 4.5 standard deviations above the mean *F*_ST_ as a cutoff (i.e., z-score > 4.5; *see* Methods), genomic regions of ~0.86 Mb were identified across 34 contigs, of which 25 overlapped with a total of 58 protein-coding genes (Fig 2B). Considering the fragmented nature of our draft reference genome, these regions may not all represent independent unlinked loci, particularly when outlier windows are located near the ends of the contigs and thereby miss flanking regions of low *F*_ST_. One important consequence of this relatively fragmented assembly is that we cannot be certain of exactly how many high *F*_ST_ outlier regions (or QTL) differentiate S_inbred_ and RS^3^, so we have focused on high *F*_ST_ SNPs that fall within these outlier regions, especially where those SNPs fall within or close to predicted genes for which a plausible case can be made for variation in or around that gene to contribute to variation in drug response. Although the size of the individual outlier regions and the number of genes annotated in each were heterogeneous, the majority of the identified regions spanned less than 100 kb (median: 35.5 kb; interquartile range: 27 kb) and harbored less than 4 genes (Fig 2A; S6 Table). Of notable exception was the 290 kb region located on Contig53, which contained 16 outlier genes. While this region may harbor multiple, spatially separated causal variants collectively resisting the gene flow from the S_inbred_ population, it is more likely that recombination has not yet substantially eliminated hitchhiking loci due to a reduced local recombination rate and/or an overall insufficient number of serial backcrosses and drug screening. Although regions containing resistance loci are expected to display higher population differentiation relative to the genomic background, non-uniform distribution of shared ancestral polymorphisms and within-population allelic diversity has likely added a layer of noise to our *F*_ST_-based introgression mapping approach. At the most fundamental level, however, mapping resolution is limited by the extent to which causal variants are decoupled from neutral hitchhiking loci, and therefore, additional rounds of backcrosses and drug screening would be expected to have helped more fully resolve causal variants from those that are closely linked. Notwithstanding these caveats, this analysis reveals an architecture of resistance genetics that is characterized by multiple regions of elevated [outlier] *F*_ST_ between S_inbred_ and RS^3^. This observation leads to the conclusion that multidrug anthelmintic resistance is likely a polygenic trait and to the hypothesis that these outlier regions of elevated *F*_ST_ represent quantitative trait loci (QTL) that are the products of selection for resistance.

**Fig 3:**
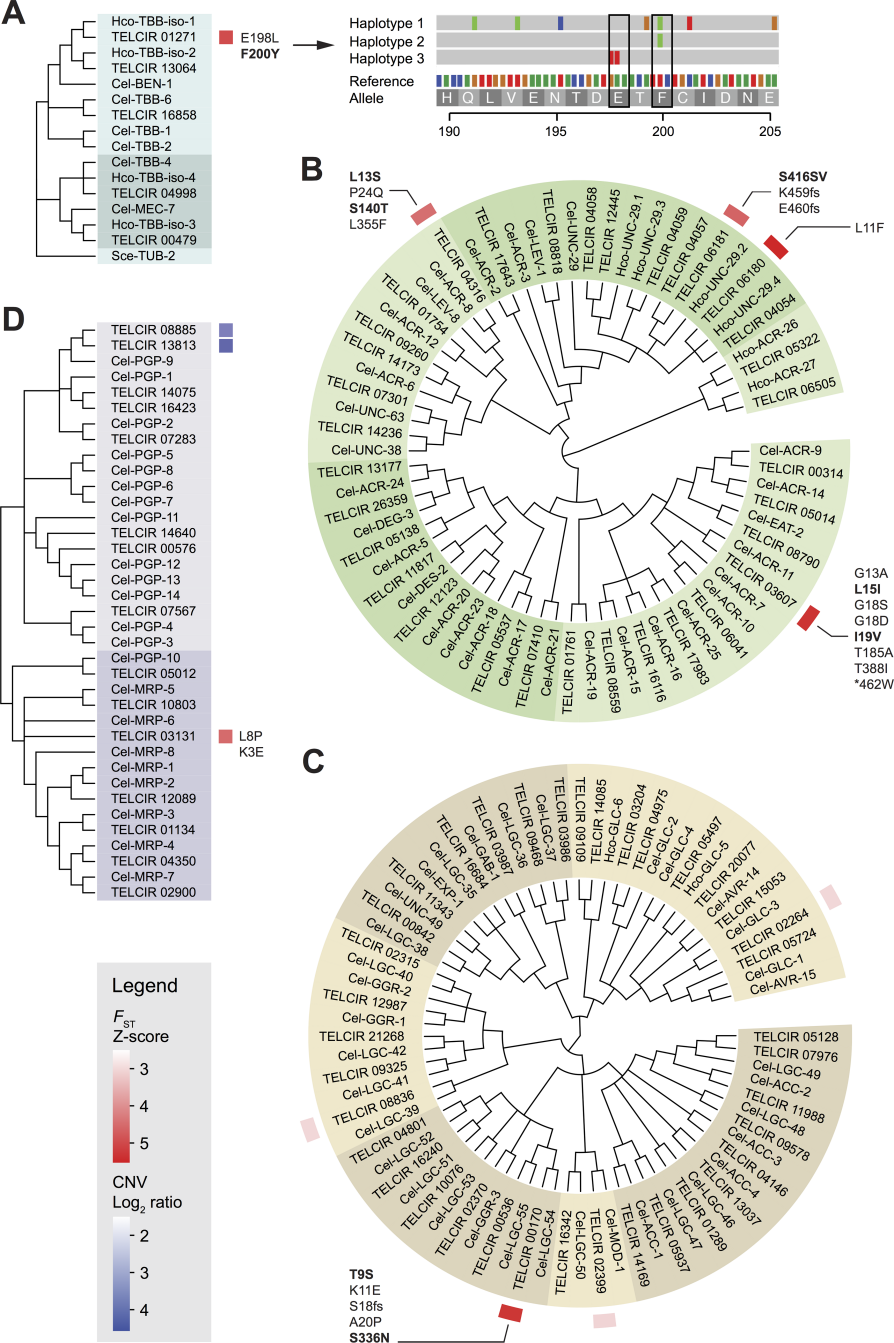
**Maximum likelihood phylogeny of (A) β-tubulins, (B) ligand-gated cation channels, (C) ligand-gated anion channels, and (D) ATP-binding cassette transporters in *Teladorsagia circumcincta*.** Unsupported nodes (bootstrap support less than 50%) were collapsed to polytomy. The shading on the trees highlights monophyletic groups. *Caenorhabditis elegans* (Cel) and *Haemonchus contortus* (Hco) homologs were included to help resolve the phylogeny. Tubulin from *Saccharomyces cerevisiae* (Sce-TUB-2) served as an outgroup. Gene-wise fixation index (*F*_ST_) and copy number variation (CNV) between the RS^3^ and S_inbred_ populations of *T*. *circumcincta* were represented as a heatmap. Non-synonymous coding variants identified in the *F*_ST_ outlier genes (z-socre > 4.5) were reported and loci with >50% allele frequency differences were indicated in bold. *De novo* assembly [101] of β-tubulin *isotype-1* (TELCIR_01271) from the RS^3^ strain revealed haplotypes harboring E198L (GAa/TTa) and F200Y (tTc/tAc) variants.

To test this hypothesis, and to help prioritize the candidate genes located in the outlier regions (Fig 2; S7 Table) for more detailed analysis, we critically evaluated whether any of these genes have known/predicted functions that can be plausibly connected to anthelmintic resistance in light of our current understanding of the mechanisms of drug action [7]. Among the most notable candidates were a β-tubulin gene (TELCIR_01271), a major target of benzimidazole anthelmintics, and putative orthologs of *Cel-unc-29* nicotinic acetylcholine receptor (nAChR) subunit (TELCIR_06180) that may constitute a component of a levamisole-sensitive receptor in *T*. *circumcincta*. Interestingly, additional members of the Cys-loop ligand-gated ion channel families (LGICs) were represented including putative orthologs of the *Cel-acr-11* nAChR (TELCIR_03607) and the *Cel-lgc-54* LGIC (TELCIR_00170). Overall, GO terms attributable to these LGICs, such as acetylcholine-activated cation-selective channel activity (*P* = 2.6 × 10^−3^), extracellular ligand-gated ion channel activity (*P* = 6.3 × 10^−3^), and postsynaptic membrane (*P* = 6.7 × 10^−3^) were significantly overrepresented among the outlier genes (Table 1). These results underscore the potential contribution of target gene variation in the development of anthelmintic resistance in *T*. *circumcincta* under field conditions, although our data do not rule out a more complex genetic architecture involving additional, presently uncharacterized genes. To further contextualize our findings in relation to previously reported anthelmintic resistance-associated genes and gene families, we examined gene-wise *F*_ST_ values and highlighted non-synonymous SNPs that showed not only significant (*P* < 1.0 × 10^−5^) but indeed substantial differentiation between the susceptible and the resistant strains (Fig 3; S7 and S8 Tables).

**Table 1:** Over-represented GO terms among genes located in *F*_ST_ outlier regions (z-score > 4.5).

Gene Ontology		Hypergeometric test *P*-value
Cellular component	Postsynaptic membrane	6.7 × 10^−3^
	Synapse part	9.8 × 10^−3^
Molecular function	Acetylcholine-activated cation-selective channel activity	2.6 × 10^−3^
	Extracellular ligand-gated ion channel activity	6.3 × 10^−3^
	Ligand-gated channel activity	7.0 × 10^−3^
	DNA-directed RNA polymerase activity	8.4 × 10^−3^
	Ion channel activity	8.8 × 10^−3^
	Channel activity	8.8 × 10^−3^

In the present genome assembly, we identified two paralogs of β-tubulin (*isotype-1* and *-2*) that are co-orthologous to *Cel-ben-1*, the locus which confers benzimidazole (BZ) sensitivity in *C*. *elegans* [22]. This finding is in line with the model of a lineage-specific duplication in trichostrongylid species [23]. Both isotypes have been implicated in BZ resistance [24, 25], with the hypothesis that selection occurs in two stages [26]: an initial reduction in diversity at *isotype-1* followed by the loss of *isotype-2*. We observed only *isotype-1* (TELCIR_01271) variation associated with the outlier loci with high *F*_ST_ (z-score > 4.5) in our genome-wide survey of the resistant backcross progeny (RS^3^). Furthermore, no evidence of selection was detected in any of the remaining members of the *T*. *circumcincta* β-tubulin gene family (Fig 3A). In the case of β-tubulin *isotype-1*, two non-synonymous coding variants, E198L (GAa/TTa) and F200Y (tTc/tAc), were exclusively found in the RS^3^ population and present at allele frequencies of 28.1% and 72.8%, respectively. The F200Y variant confers BZ resistance in *H*. *contortus* [27], and has been widely recognized in many species of parasitic nematode as a major resistance determinant. Although amino acid substitutions at position 198 (e.g., E198A) are less common in nematodes, variants at this position have been linked to BZ resistance phenotypes in *H*. *contortus* [28] and, more recently, in *T*. *circumcincta* [29], and molecular modeling suggests that the associated loss of hydrogen bonding interactions may play a role in the resistance mechanism [30]. We reconstructed three segregating haplotypes of β-tubulin *isotype-1* in the RS^3^ population over the exonic region harboring E198L and F200Y variants (Fig 3A; S1 Fig). The inferred haplotype structure indicates that (i) F200Y occurs on at least two distinct and diverse haplotype backgrounds, suggesting multiple independent origins of the variant allele, and (ii) E198L and F200Y variants occur in *trans* on separate haplotypes. In agreement with this haplotype reconstruction, genotyping of individual male worms from the S_inbred_ (n = 94) and RS^3^ (n = 79) populations failed to detect any individuals homozygous for both resistance alleles (R_198_R_198_/R_200_R_200_) although worms homozygous resistant at one locus only (S_198_S_198_/R_200_R_200_ and R_198_R_198_/S_200_S_200_) were observed, as were double heterozygotes (S_198_R_198_/S_200_R_200_) (S2 Fig; S9 Table). Considering that S_198_R_200_ haplotype was segregating in the RS^3^ population at a minimum inferred frequency of 79.7% (S9 Table), the absence of single heterozygotes (especially worms heterozygous for only P198) was consistent with (and also supported) the conclusion of our Pool-seq analysis that R_198_R_200_ haplotype was not present in the RS^3^ population. Sequencing of *isotype-1* from the same individuals confirmed the existence of multiple haplotypes, supporting the conclusion that BZ resistance conferring alleles arose several times in the OR parent of RS_3_ (S3 Fig), as has been reported for BZ resistance conferring alleles in the UK [29]. Although further work will be necessary to fully determine the extent to which E198L contributes to the overall resistance phenotype, the absence of haplotype(s) simultaneously harboring both variants suggests that, under field conditions, E198L can confer BZ resistance (and hence was selected) independently of F200Y, and that β-tubulin carrying both variants either is detrimental to organismal fitness (i.e., negative intramolecular epistasis) or the double mutation event (or recombination over an interval of <6bp) required to give rise to a *cis* haplotype is sufficiently unlikely that it is not observed.

Cholinergic anthelmintics, such as LEV and pyrantel, induce spastic neuromuscular paralysis by selectively opening nAChRs, a family of pentameric ion channels belonging to the Cys-loop LGIC superfamily that convert neurotransmitter binding into membrane electrical depolarization. Each receptor subunit has an N-terminal extracellular ligand-binding domain (ECD) followed by four transmembrane helices (TMD) that form the ion channel [31]. Nematode genomes encode a large number of nAChR subunits (~30) [32] and different subunit combinations result in pharmacologically distinct receptors [33]. Although the precise subunit composition of LEV-sensitive nAChR in *T*. *circumcincta* has not yet been determined, a putative model for trichostrongylid species suggests a likely involvement of parasite orthologs of *Cel-unc-29*, *Cel-unc-38*, *Cel-unc-64* and *Cel-acr-8* in receptor formation [33], and mutation, truncation and decreased expression of these subunits have been observed in field-selected LEV-resistant trichostrongylids [34–36]. *Tci-unc-29*.*4* and *Tci-acr-11* nAChR genes were identified in our *F*_ST_ outlier analysis and, in addition, the genomic regions encoding *Tci-unc-29*.*2* (TELCIR_06181) and *Tci-acr-8* (TELCIR_04316), displayed relatively high *F*_ST_ values (z-score of 4.32 and 4.18, respectively) (Fig 3B). Within these subunit genes, 16 non-synonymous coding variants were found. When considering the potential consequences of the substitutions based on the amino acid properties [37] and their locations relative to the ligand-binding or the transmembrane domains, the probability that any of these variants have drastic detrimental effects on protein structure and function appears to be relatively low. This observation is consistent with the view that, under field conditions, loss of function variants are likely to experience negative selection due to reduced fitness. Several of the variants (e.g., T388I and *462W (stop loss) in *Tci-acr-11* and P24Q in *Tci-acr-8*) appear to have a greater potential to alter protein function, although the allele frequency of these variants in the resistant population is not substantially different from that in the susceptible population, suggesting that they are unlikely to play a direct role in LEV resistance (S8 Table). Further biochemical, pharmacological and structural modeling work will be necessary to fully assess and understand the impact of these alleles on drug-target interactions. These results also raise the possibility that drug selection may have acted primarily on the noncoding regulatory variants of the *F*_ST_ outlier nAChR genes. A preliminary assessment of the transcript abundance levels (*see* Methods) suggested that *Tci-acr-11* transcript was substantially less abundant in the resistant strain (RS^3^) relative to the susceptible strain (S_inbred_) (RS^3^/S_inbred_ log_2_ ratio: -5.02; *see* S7 Table), in a manner similar to previous reports that showed decreased expression of nAChR subunit genes in various LEV-resistant trichostrongylid populations [38–40]. However, because of the limited mapping resolution of the present study and our generally poor understanding of the functional consequences of noncoding variants, we are unable to identify specific candidate noncoding mutation(s) that could contribute to the resistance phenotype. Furthermore, although mutant screens in *C*. *elegans* indicate that genetic variations in calcium-mediated muscle contraction signaling pathway and ancillary proteins involved in nAChR assembly/maintenance may influence LEV susceptibility [41, 42], we did not observe any significant evidence of genetic differentiation among the genes implicated in the LEV excitation-contraction pathway in the RS^3^ population (S7 Table).

The predicted gene TELCIR_00170 (*Tci-lgc-54*) is one of the top *F*_ST_ outliers in our analysis (S7 Table). It belongs to the Cys-loop ligand gated chloride channel branch of the LGIC superfamily and is distinct from the nAChRs associated with LEV resistance (Fig 3C) and from GluCl and GABA receptor family members. The likely *C*. *elegans* ortholog, *Cel-lgc-54*, is described as a predicted “ligand unknown” biogenic amine-gated chloride channel [43] and as a GABA-receptor [44] but has not been implicated previously in relation to IVM resistance. Although the ligand for nematode LGC-54 is not yet identified, the predicted protein contains a tryptophan in ligand-binding loop C (amino acid position 231), which has been hypothesized to be a key residue for binding amines [45], and it is known that other family members (including *Cel-lgc-55*, the most closely related paralog in *C*. *elegans*) are activated by serotonin, dopamine and tyramine [46–48]. Furthermore, a gene encoding a putative dopamine receptor, *Hco-ggr-3*, has been implicated in IVM resistance in *H*. *contortus* [48]. In the RS^3^ population, we identified 5 non-synonymous variants in *Tci*-*lgc-54*: T9S, K11E, S18fs, A20P, and S336N. The former four are located upstream of the N-terminal ECD, and the latter is located at the beginning of the cytosolic loop between the third (M3) and the fourth (M4) TMD alpha helices. Notably, the frameshift variant at position 18 introduces a premature stop codon and the A20P variant located at the predicted signal peptide cleavage site (position 20–21) has a potential to interfere with the proper signal peptide processing (S4 Fig). Failure of signal peptide cleavage is likely to result in mislocation and/or degradation of the protein and thus behave as a loss-of-function mutation. It is of interest in this context that (a) large deletion alleles of *Cel-lgc-54* and *Cel-ggr-3* in *C*. *elegans* are viable suggesting that these channel subunits are not essential (although loss-of-function mutations in *Cel-lgc-55* confer subtle behavioral phenotypes [47]), (b) reduced IVM sensitivity in *H*. *contortus* is associated with reduction in the transcript abundance of *Hco-ggr-3* [48] and (c) a four-amino-acid deletion in the N-terminal region of *Cel-glc-1* has been linked to IVM resistance in *C*. *elegans* [49].

Although glutamate-gated chloride channels (GluCls) are considered its main targets, IVM may also interact directly with other anionic Cys-loop LGICs, including GABA_A_ and glycine receptors [50, 51] and irreversible activation of these inhibitory chloride channels by IVM results in flaccid paralysis and eventual expulsion of the parasite [52, 53]. Several glutamate and GABA gated chloride channel genes have been implicated in IVM resistance in *H*. *contortus* (e.g. Beech et al. 2013) but none of these genes showed significant values for *F*_ST_ in our analysis of *T*. *circumcincta*. In addition to genes involved directly or indirectly in neurotransmitter functions, genes putatively responsible for amphid neuron defects in *C*. *elegans* and *H*. *contortus*, such as *Cel-che-3*, *Cel-dyf-7* and *Hco-dyf-7*, have been implicated in IVM resistance in these species [54–56]. Again, none of the likely *che-3* and *dyf-7* orthologs in *T*. *circumcincta* displayed notably high *F*_ST_ values in our analysis (S7 Table). Although it is possible that different IVM resistance mechanisms are involved in different nematode species, in a recent study of UK field populations of *H*. *contortus*, no evidence of selection by IVM was detected for *Hco-lgc-37*, *Hco-glc-5*, *Hco-avr-14* or *Hco-dyf-7* [57]. Furthermore, a recent analysis of IVM resistant backcross populations in *H*. *contortus* has also suggested that these candidate genes were not associated with resistance [58]. It is thus conceivable that some of the putative candidate genes from earlier single-locus studies may represent false-positive associations. If, as we conclude on the basis of the data reported here, multidrug anthelmintic resistance is a polygenic quantitative trait, one explanation for this apparent discrepancy is that in the resistant population that we analyzed these genes are not under selection. The observation that >1 genotype can result in the same phenotype is expected for quantitative traits. This may also, for example, explain why macrocyclic lactone resistance in particular seems so genetically heterogeneous, with many different candidates apparently under selection in different resistant populations.

### Copy number variations associated with multiple-anthelmintic resistance

We also examined copy number variations (CNVs) between the RS^3^ and S_inbred_ strains of *T*. *circumcincta* (Fig 2C). Since our reference assembly was generated using the S_inbred_ strain, we focused our analysis on genomic regions displaying increased copy number in the RS^3^ population relative to the S_inbred_ population, because any copy number decrease in the RS^3^ strain was likely to have been confounded with a potential mapping bias against highly divergent reads containing non-reference alleles (especially in the intronic and intergenic regions). The top 10 protein-coding CNV regions showing the most extreme inter-strain variation (log_2_ read count ratio > 2.9) contained 13 genes, 4 of which are likely orthologs of *C*. *elegans* P-glycoprotein 9 (*Tci-pgp-9*; TELCIR_08885, TELCIR_13813, TELCIR_19247 and TELCIR_19884) (Table 2; Fig 3D). These sequences (one complete and three partial genes) appear to represent the individual haplotypes of *Tci-pgp-9* that are segregating in the S_inbred_ population. While our pooled sequencing data provide strong evidence of an increase in *Tci-pgp-9* copy number on average in the resistant population relative to the susceptible population, it remains challenging to reliably resolve the full haplotype sequences and their respective within-population copy number variability for each population. Nevertheless, confirmation that RS^3^ strain parasites carry additional copies of this gene compared to S_inbred_ parasites was provided by a separate investigation of *Tci-pgp-9* copy number using single worm genomic DNA quantitative PCR (S5 Fig). Furthermore, these single worm data showed that certain *Tci-pgp-9* haplotypes (i.e., allelic variants defined on the basis of the first inter-nucleotide binding domain (IBDA) sequence polymorphisms) (S6 Fig) occurred only in worms exhibiting increased *Tci-pgp-9* copy number (IBDA haplotypes 3, 6 and 10) (S10 Table), suggesting (a) that these haplotypes arose as a result of the gene duplication event(s) that gave rise to the increase in copy number, (b) that the duplication(s) occurred long enough ago that the duplicated copies have started to diverge and/or (c) selection for or against functional differences in IVM-affinity conferred by specific haplotypes. One further haplotype that appeared to be enriched in the RS^3^ population, haplotype 2, does not occur in worms that carry additional copies of *Tci-pgp-9* (S10 Table), suggesting that selection for this haplotype in resistant worms is not related to increased copy number.

**Table 2:** Top 10 CNV regions that contain protein-coding genes in *Teladorsagia circumcincta* RS^3^ strain showing the highest level of copy number increase relative to the S_inbred_ strain.

CNV region	Log_2_ ratio (RS^3^/S_inbred_)	Overlapping gene	
Cont17796:492–5412	8.8	TELCIR_20472	Conserved domain protein
		TELCIR_20473	Hypothetical protein
Cont4607:20664–27060	7.2	TELCIR_13581	Conserved domain protein
Cont13316:492–8364	6.1	TELCIR_19247	ABC transporter (*Tci-pgp-9*)
Cont196631:492–2460	6.0	TELCIR_23120	Hypothetical protein
Cont15521:492–3936	4.8	TELCIR_19884	ABC transporter (*Tci-pgp-9*)
Cont5:324720–326196	4.4	TELCIR_00177	Conserved hypothetical protein
Cont204265:492–1968	3.3	TELCIR_23838	Conserved hypothetical protein
Cont4815:28044–30504	3.3	TELCIR_13812	Hypothetical protein
		TELCIR_13813	ABC transporter (*Tci-pgp-9*)
Cont1731:492–7872	2.9	TELCIR_08884	Hypothetical protein
		TELCIR_08885	ABC transporter (*Tci-pgp-9*)
Cont1090:71832–80196	2.9	TELCIR_07060	Hypothetical protein

P-glycoprotein (P-gp) is an ATP-binding cassette (ABC) transporter with two homologous halves, each containing a TMD and a cytoplasmic nucleotide-binding domain (NBD). Using the energy from ATP hydrolysis, P-gp actively transports many lipophilic compounds (both endogenous metabolites and xenobiotics) out of the cell from the inner leaflet of the membrane, providing a mechanism by which anthelmintic concentration at the receptor site may be reduced. Sequence polymorphism and constitutive or inducible overexpression of P-gp’s have been reported in IVM-resistant populations of several nematode species, including *T*. *circumcincta*, in support of the hypothesis that an increased drug efflux due to changes in expression, activity and/or substrate specificity of ABC transporters can contribute to IVM resistance [59, 60]. Our preliminary assessment (by RNA-seq) indicates that *Tci-pgp-9* transcripts are more abundant in the RS^3^ population relative to the S_inbred_ population (log_2_ ratio > 3) (S7 Table), suggesting that the *Tci-pgp-9* copy number increase facilitates (constitutive or inducible) increased expression of the transporter in the resistant population. Cloning and sequencing of individual cDNA transcripts corresponding to the N- and C-terminal transmembrane domains (with their extracellular loops) showed that the predominant transcripts in the RS^3^ population carry either a splice variant that results in a deletion of 45 aa from the first predicted extracellular loop between TM1 and TM2 (a region of the protein hypothesized to play a role in substrate binding), or a full length variant that contains 3 non-synonymous amino acid substitutions in the same predicted loop (S7–S9 Figs). Thus, it appears likely that a combination of increased expression (via increased copy number) and sequence polymorphism may contribute to the association between *Tci-pgp-9* and IVM resistance in the RS^3^ strain.

### Mapping of genetic loci associated with ivermectin resistance based on single worm genotyping

The design of our introgression strategy, which aimed to identify genomic regions concurrently selected in response to BZ, LEV and IVM, did not allow us to directly assess the relative contribution of candidate loci to resistance against each of the individual anthelmintic classes. We therefore undertook a complementary mapping approach with a specific focus on IVM resistance using F2 populations derived from a cross between the S_inbred_ susceptible and the R_par_ multiple-anthelmintic resistant isolates, i.e., the parental strains of the RS^3^ backcross progeny population on which our whole-genome introgression study was based (Fig 1A). Individuals from IVM-screened and drug-naïve F2 mapping populations (n = 24 male worms from each group) were genotyped by ddRAD-seq, a reduced-representation genome sequencing method, yielding a total of 0.59 million variant calls. Using the *F*_ST_ estimates of segregating SNPs that satisfied a minimum sampling depth of 10 individuals in both populations (n = 2,628) (S11 Table), we identified outlier loci and linked genes (i.e., contigs) most strongly differentiated in the IVM-survivor group relative to the drug-naïve control group, and compared the outcome against the outlier genes identified from the introgression mapping experiment. Even though there was a high rate of allele dropout (most likely due to sequence polymorphism within the restriction sites in our mapping population leading to the loss of affected restriction fragments from our RAD-seq libraries), we were able to survey part of the genome (349 contigs; combined length = 57.6 Mb or ~8% of the genome) for outlier loci. It has been shown that, in RAD-seq experiments, missing data can inflate *F*_ST_ values and rates of false-positive outliers increase as the chromosome sampling depth cutoff decreases [61]. We indeed observed a strong dependency between the variance of *F*_ST_ estimates and the allele dropout (S10 Fig) and therefore determined outliers in each individual sampling depth category separately under the assumption that outliers were evenly distributed across loci irrespective of missing data (Fig 2D).

Within the part of the genome that was subjected to *F*_ST_ outlier analysis, we identified 18 genes across 5 contigs that displayed some evidence of genetic differentiation in both the introgression and F2 mapping experiments (i.e., minimum *F*_ST_ z-score of 2.5 in both datasets) (S12 Table). Included in this combined list is *Tci-lgc-54*, one of the top outlier genes from the genomic introgression analysis. Although the evidence of selection is not as strong in the IVM-survivor F_2_ mapping population as it was in the introgressed multiple-anthelmintic resistant population (RS^3^), the amine-gated chloride channel *Tci-lgc-54* is the only candidate LGIC that is supported strongly by both of the mapping approaches (S13 Table) and thus merits further analysis as a potential IVM resistance gene in *T*. *circumcincta*. It is important to note that the individual F2 generation IVM treatment survivors analyzed here were not significantly more resistant to either BZ or LEV treatment as a result of the IVM treatment (*see* S14 and S15 Tables). Thus the resistance phenotypes appeared to have segregated independently, indicating that *Tci-lgc-54* was an *F*_ST_ outlier in IVM-resistant F2 segregants that remained susceptible to BZ and LEV treatment. Consequently, despite strong evidence for selection of ABC transporters, these data do not support a single “multidrug resistance mechanism” able to confer resistance simultaneously to all 3 drug classes.

We were unable to assess whether the *Tci-pgp-9* revealed by Introgression analysis as a strong candidate IVM-resistance locus co-segregates with IVM resistance in the F2 mapping population because of the absence in the RAD-seq data of linked SNP makers with adequate sampling depth for *F*_ST_ outlier analysis. A different ABC transporter, *Tci-mrp-6* (TELCIR_03131) (Fig 3D), is however present on the combined introgression/segregation list. Our results suggest the interesting possibility that multiple ABC transporters may be involved in IVM efflux, either within the same worm or in different worms, in the RS^3^ multiple-anthelmintic resistant population. In support of this conclusion, several reports in other parasitic nematodes implicate a range of ABC transporter family members [62–64], implying that there may be many combinations of ABC transporters able to contribute to IVM-resistance.

A novel, strongly-supported, candidate region in our combined outlier list (S12 Table) is on Contig209, which contains two putative triacylglycerol lipase genes (TELCIR_02985 and TELCIR_02988; *F*_ST_ z-score > 3.3). Intriguingly, a triacylglycerol lipase/cholesterol esterase gene (F54F3.3) has been shown in *C*. *elegans* to respond transcriptionally to IVM exposure [65], although it remains to be determined whether the lipase activity plays a role in a drug-induced starvation-related stress response that facilitates tolerance of or recovery from ivermectin toxicity, or whether lipid metabolism may play a more direct role in IVM metabolism or detoxification. It is of interest to note that a similar pool sequencing analysis of ivermectin response in *Onchocerca volvulus* [66] points to the involvement of likely orthologues of these genes in a distantly related nematode parasite of significant medical importance.

The work reported here is the first to combine classical genetic methods such as introgression and segregation analysis with new genomic tools such as RAD-seq and whole genome re-sequencing to analyze multiple-anthelmintic resistance in a parasitic nematode. The nematode species examined, *T*. *circumcincta*, is an economically significant, globally distributed gastrointestinal parasite of small ruminants. More importantly however, with the rapid proliferation of mass drug administration programs globally for treatment of helminth infections of humans, there is an urgent need to better understand the genetic basis of resistance to the drugs that form the basis of those programs. We show clearly that resistance to each of the three drug classes segregates independently of the others and that for LEV and IVM resistance in particular, multiple loci likely contribute to the resistance in a variety of ways (possible reduction/modulation in target site sensitivity, reduced target site expression, increased drug efflux, etc.), so that drug resistance in these parasites should best be thought of as a multifactorial quantitative trait rather than a simple, discrete Mendelian character. The polygenic genetic architecture of resistance provides an explanation for the apparent discrepancies between the many single, candidate gene studies and, since many genes can contribute to resistance, it seems likely therefore that the combination selected in any given circumstance is as likely to be a product of genetic drift as of selection *per se*. Furthermore, it is also clear from these data that alleles that contribute to resistance for each drug class have arisen many times on different genetic backgrounds, giving rise to a heterogeneous mix of “resistance haplotypes” that implies soft rather than hard selective sweeps. This is most obvious at the β-tubulin *isotype-1* locus. Although selection at this locus appears to be necessary and sufficient for BZ-resistance, we observed extensive polymorphism surrounding the amino acid 198 and 200 determinants of resistance, suggesting soft selection from multiple pre-existing variants at P198 or P200 rather than hard selection of a single allele at a single position has occurred for this resistance. Similar genetic heterogeneity at this locus has been observed in other BZ-resistant isolates of *T*. *circumcincta* and *H*. *contortus* [29].

In conclusion, this work elucidates the polygenic, quantitative trait genetic architecture of multiple anthelmintic resistance in a parasitic nematode for the first time and establishes a framework on which future studies of the inevitable evolution of anthelmintic resistance in nematode parasites of humans can be based. In this context, it is significant that a similar study of ivermectin response in *O*. *volvulus* [66] points to a similar genetic architecture, with hits either to likely orthologues of genes identified here or to similar neuronal functions, thus demonstrating the utility of studies in more tractable parasite species such as *T*. *circumcincta*.

## Materials and methods

### Ethics statement

All experimental procedures used in generating the parasite material for this study were approved by AgResearch’s Wallaceville Animal Research Centre Animal Ethics Committee under the Animal Welfare Act 1999 in New Zealand [AEC application numbers 516, 562 & 636].

### Parental isolates used in developing the introgressed strain

The multiple-anthelmintic resistant field strain of *T*. *circumcincta* (OR strain) used in this study was isolated in New Zealand in 1996 from lambs which had been grazing a property previously occupied by Angora goats (Leathwick DM, personal communication). Fecal nematode egg count reduction tests undertaken on the lambs revealed that none of the three broad-spectrum anthelmintic families available at that time, i.e., oxfendazole (BZ), levamisole (LEV) and ivermectin (IVM), were fully effective against this isolate. Prior to inter-strain crosses being set up, a population of OR was maintained for five generations in pen-raised goat kids, and screened at each generation with selected representatives of BZ (Systamex; Schering Plough, Kenilworth, NJ; 4.5 mg/kg), LEV (Levicare; Ancare New Zealand, Auckland, New Zealand; 7.5 mg/kg) and IVM (Ivomec liquid for sheep and goats, Merial New Zealand; 0.2 mg/kg) to maximize the proportion of worms homozygous for resistance to each of them. Anthelmintics were administered to the goat kids at the manufacturer’s recommended dose rate unless no specific goat dose rate was provided, in which case a standard sheep dose rate was used (as was common practice on goat farms in New Zealand before the widespread emergence of anthelmintic resistance in this host species). The efficacy of all these drugs, used either individually or in combination, was very low against the resulting resistant parental (R_par_) strain (Fig 1B). The anthelmintic susceptible S strain of *T*. *circumcincta* was originally isolated from field-grazed lambs in New Zealand during the 1950’s prior to the widespread use of broad-spectrum anthelmintics (Elliott DC, personal communication). This isolate had subsequently been maintained at Wallaceville Animal Research Centre (AgResearch, New Zealand) by annual passage through pen-raised drug-naïve lambs. Given that significant genetic diversity can be maintained even in laboratory-passaged nematode populations of limited size, the S isolate was subjected to two generations of half-sib mating in an attempt to reduce the background genetic variance in preparation for introgression mapping. Briefly, mature eggs were collected from the oviduct of a single gravid adult female recovered from the host’s abomasum, and cultured to collect infective larvae. Thirteen of these sibling larvae were used to orally infect a pen-raised parasite-free goat kid, and half of the resulting progeny were used to re-infect the same host to supplement the existing infection. A second goat kid was subsequently infected using larvae cultured from the first. Sibling mating was repeated as before by isolating and culturing eggs from a single adult female worm isolated from the abomasum of the second kid. Twenty sibling larvae from this culture were used to infect a third worm-free goat kid whose fecal output was cultured for subsequent infections. Anthelmintic efficacy testing on this partially inbred S strain (S_inbred_) revealed that representatives of all three broad-spectrum anthelmintic classes were highly effective (efficacy >99.0% for BZ, LEV, and IVM) [15].

### Introgression of multiple-anthelmintic resistance genes into an inbred anthelmintic susceptible genetic background by serial backcrossing

A schematic of the backcrossing and selection experiment is outlined in Fig 1A. Crosses between R_par_ and S_inbred_ strains of *T*. *circumcincta* were performed by surgical transfer of worms from separate donor goat kids (containing either R_par_ or S_inbred_ worms) into the abomasum of a recipient goat kid. In order to ensure that the female S_inbred_ worms had not yet mated, infection of the donor kids was timed so that the females would be 10 days old and thus still at the late-fourth developmental stage (L_4_) at the time of transfer, while male R_par_ worms would be 5 weeks old and thus adults. Ten days before transfer, the R_par_ worms were screened with BZ, LEV and IVM. Worms were collected from donor goat abomasa, rinsed with phosphate-buffered saline over a 45μm Endecott sieve, and inspected microscopically. Approximately 300 male adult male (R_par_) and an equivalent number of L_4_ female (S_inbred_) worms were collected into a modified Nematode Growth Medium containing 0.4% w/v agar [67], and surgically transferred into the abomasum of a previously worm-free recipient goat kid. The [heterozygous] F_1_ progeny resulting from this cross were then used to infect another worm-free kid to obtain an F_2_ generation in which the alleles for anthelmintic resistance were expected to have segregated. The F_2_ infective larvae cultured from this kid were used to infect a further worm-free goat kid, which was then subjected to successive doses of IVM, BZ and LEV over a period of 24 hrs at 28 days post-infection so that the F_3_ generation would be derived from worms carrying the full complement of genes needed for multiple-anthelmintic resistance. In order to maximize the frequency of resistance alleles in the population, F_3_ worms were passaged for a further two generations of drug screening with IVM, BZ and LEV. At this point a backcross between the anthelmintic-screened F_5_ generation and S_inbred_ worms was set up using similar procedures to those described for the initial crosses. F_2_, F_3_ and F_4_ generations of the backcross worms were then each screened, as before, with all 3 anthelmintic classes before a final round of backcrossing and a further 4 generations of drug screening. Because each generation of backcrossing reduces the proportion of the donor parent genome present in the population by half, the resultant multiple-anthelmintic resistant worm population (RS^3^) was expected to have a genetic makeup largely similar (7/8) to that of the susceptible recurrent parent (S_inbred_) but, at the same time, be carrying the anthelmintic-resistance genes derived from the R_par_ strain. Crosses based on mass mating and multiple generations of drug selection (between and after backcrosses) were an important design feature to create variation in recombination breakpoints and divide individual introgression blocks (i.e., segments of DNA of R_par_ origin untouched by recombination) into smaller fragments.

### Preparation of genomic DNA and total RNA for high-throughput sequencing

Parasite-free lambs, maintained indoors on a diet designed to avoid any unintended nematode infections, were each infected with approximately 24,000 larvae of either the RS^3^ strain (2 lambs) or the S_inbred_ strain (3 lambs) of *T*. *circumcincta*. At 28 days post-infection, lambs that had received the RS^3^ strain were treated successively with IVM, BZ and LEV over a period of 24 hrs. At 37 days post-infection (9 days post-treatment), the RS^3^ worms were collected from the abomasum, washed free of all debris in physiological saline, and then transferred in mixed sex batches of 300–500 individuals into 1.5 ml tubes in which they were snap frozen at -80°C. The S_inbred_ worms were similarly collected and snap frozen at 28 days post-infection without anthelmintic treatment. Genomic DNA was isolated from each of the strains using a method modified from that described by Sulston and Hodgkin [68]. Each worm sample (after thawing) was suspended in 150–200 μl of lysis solution [100 mM NaCl, 100 mM Tris-HCl (pH 8.5), 50 mM EDTA (pH 7.5), 1% SDS, 2% β-mercaptoethanol and 200 μg/ml proteinase K], placed in a glass/teflon tissue grinder and thoroughly homogenized. The homogenate was transferred into a sterile 10 ml tube and supplemented with additional lysis solution to a total volume of 3 ml. After incubation at 65°C for 16 hr with gentle mixing, 10 μl of RNase A (100 mg/ml; QIAgen, Hilden, Germany) was added and the lysate incubated for 10 min at 45°C. To remove protein/polysaccharide complexes (which can be problematic in nematode DNA preparations), 750 μl 5M NaCl and 500 μl CTAB/NaCl (10% CTAB in 0.7M NaCl) were added, and after gentle mixing the tube was incubated for 15 min at 65°C. The lysate was then extracted successively with equal volumes of phenol/chloroform/isoamyl alcohol (25/24/1) and chloroform/isoamyl alcohol (24/1). Following recovery of the aqueous layer from the final extraction, DNA was precipitated by the addition of 2 volumes of absolute ethanol (4°C), pelleted by centrifugation, washed twice in 70% ethanol (4°C), briefly air-dried, and re-suspended in 150 μl 10mM TrisCl (pH 8.5). Total RNA was also prepared from the mixed-sex batches of adult *T*. *circumcincta* from each of the S_inbred_ and RS^3^ strains. Frozen worm samples were added to 1 ml of TRIzol reagent (Invitrogen, Carlsbad, CA) and the mixture ground to a powder in liquid nitrogen in a mortar and pestle. The resulting powder was transferred into a microfuge tube, 200 μl chloroform was added and the tube was shaken vigorously before being centrifuged at 12,000g for 15 min at 4°C. Following recovery of the upper phase, the RNA was precipitated by the addition of 500 μl of isopropanol and pelleted by centrifugation at 12,000g for 10 min at 4°C. The RNA pellet was then washed in 75% ethanol and air-dried briefly before re-suspending in 40 μl UltraPure DNase/RNase Free Distilled Water (Invitrogen). The integrity and yield of the RNA was verified using the Bioanalyzer 2100 (Agilent Technologies, Cedar Creek, TX).

### Sequencing, *de novo* assembly and annotation of an anthelmintic-susceptible *T*. *circumcincta* reference genome

As a basis for introgression mapping and comprehensive variant analysis, we generated a draft genome sequence for the anthelmintic-susceptible S_inbred_ strain of *T*. *circumcincta* (BioProject ID: PRJNA72569). Whole genome shotgun libraries (fragments and mean insert size of 3kb and 8kb) were generated as previously described [69] and sequenced using a Genome Sequencer Titanium FLX (Roche Diagnostics, Basel, Switzerland) platform (S16 Table), and assembled using Newbler v. 2.6 [70]. To improve scaffolding, an in-house tool CIGA (Cdna tool for Improving Genome Assembly) was used to map 454 cDNA reads using BLAT [71] to the genomic assembly to link genomic contigs. Gaps were then closed using Pygap, an in-house tool, which utilizes the Pyramid assembler and uses Illumina paired-end reads to close gaps and extend contigs. The repeat library was generated using Repeatmodeler (http://repeatmasker.org), and Tandem Repeat Finder [72] was used in addition for sequence annotation. Repeats and predicted RNAs were then masked using RepeatMasker (http://repeatmasker.org). The ribosomal RNA genes were identified using RNAmmer [73] and transfer RNAs were identified with tRNAscan-SE [74]. Non-coding RNAs, such as microRNAs, were identified by sequence homology search of the Rfam database [75]. Protein-coding genes were predicted using a combination of *ab initio* programs Snap [76], Fgenesh [77] and Augustus [78] and the annotation pipeline tool Maker [79] which aligns mRNA, EST and protein information from the same species or cross-species to aid in gene structure determination and modifications. A consensus gene set from the above prediction algorithms was generated, using a previously described, logical, hierarchical approach [69]. In summary, the following Quality Index (QI) criteria were calculated: i) length of the 5’ UTR; ii) fraction of splice sites confirmed by an EST alignment; iii) fraction of exons that overlap an EST alignment; iv) fraction of exons that overlap EST or protein alignments; v) fraction of splice sites confirmed by a SNAP prediction; vi) fraction of exons that overlap a SNAP prediction; vii) number of exons in the mRNA; viii) length of the 3’ UTR and; ix) length of the protein sequence produced by the mRNA, and then the following decision making steps were followed: a) genes were screened for overlaps (<10% overlap was allowed); b) if QI[ii] and QI[iii] were greater than 0, or QI[iv] was greater than 0, then the gene was kept; c) the gene was BLASTed against Swissprot [80] (E < 1e-6). If there was similarity to a Swissprot entry, then the gene was kept; d) RPSBLAST was ran against Pfam [81] (E < 1e-3). If there was similarity to a Pfam entry, then the gene was kept; e) RPSBLAST was run against CDD [82] (E < 1e-3 and coverage > 40%). Genes that met both cut-offs were kept and f) if no hit was recorded, then a sequence similarity-based search was run against GenesDB from KEGG [83], and genes with at least a 55% identity and a bit score of 35 or higher were kept. Genes of interest (discussed in the paper) underwent manual evaluation and improvements. Gene product naming was determined by BER (http://ber.sourceforge.net). Functional domains and Gene Ontology (GO) terms were assigned using InterProScan (v. 4.8) [84]. Genes were mapped to KEGG Orthology (KO) groups using wu-blastp (E < 1e-5). Proteins with signal peptides and transmembrane topology were identified using the Phobius web server [85], and non-classical secretion was predicted using SecretomeP 1.0 [86]. CEGMA (v.2.4) [18] was used to assess the completeness of the genome without excluding partial matches. MEGA6.06 [87] was used to estimate maximum likelihood phylogenies (LG+G model).

### RNA sequencing and analysis

The same RNA samples were used to generate both Roche/454 and Illumina cDNA libraries. Both data types were used for genome annotation and the Illumina reads were used for differential expression analysis. Non-normalized oligo dT libraries for Roche/454 were generated as previously described [88]. The Roche/454 library was sequenced using a Genome Sequencer Titanium FLX (Roche Diagnostics) and the ‘sffinfo’ program was used to extract information from the SFF files. Adaptor sequences were trimmed from the sequenced reads using the 'seqclean’ software and host and bacterial contamination was removed using Newbler’s ‘gsmapper’. For the Illumina RNA-seq library construction, total RNA was treated with Ambion Turbo DNase (Ambion/Applied Biosystems, Austin, TX) and 1μg of the DNAse treated total RNA was poly(A) selected using the MicroPoly(A) Purist Kit according to the manufacturer's recommendations (Ambion/Applied Biosystems). One ng of the mRNA isolated was used as the template for cDNA library construction using the Ovation RNA-seq (v.2) kit according to the manufacturer's recommendations (NuGEN Technologies, San Carlos, CA). Non-normalized cDNA was used to construct multiplexed Illumina paired-end small fragment libraries as previously described [69], according to the manufacturer's recommendations (Illumina, San Diego, CA) with the following exceptions. In summary, 500 ng of cDNA was sheared using a Covaris S220 DNA Sonicator (Covaris, Woburn, MA) to a size range of 200-400bp. Four PCR reactions were amplified to enrich for adaptor ligated fragments and index the libraries. The final size selection of the library was achieved by an AMPure paramagnetic bead (Agencourt, Beckman Coulter Genomics, Beverly, MA) cleanup, targeting 300-500bp. To produce cluster counts appropriate for the Illumina sequencing, the concentration of the library was determined by qPCR according to the manufacturer's protocol (Kapa Biosystems, Woburn, MA). The Illumina HiSeq 2000 platform was used for generation of sequences of 100bp from samples of pooled individuals of 300–500 RS^3^ or S_inbred_ adult worms (S16 Table). RNA-seq reads were aligned to the S_inbred_ reference assembly of *T*. *circumcincta* using STAR aligner (v.2.3.0) [89] with default parameters, following the 2-pass method [90]. Transcript abundance levels were expressed in FPKM (fragments per kilobase of exon per million fragments mapped). To infer rankings of differentially expressed genes according to their effect size, GFOLD (generalized fold change) algorithm was used with default parameters [91]. Although GFOLD provides a more consistent and biologically meaningful approach to ranking differentially expressed genes than other methods for single replicate RNA-seq experiments, an analysis of variation between replicate samples is necessary to draw sound conclusions, especially on the individual gene level. The differential expression patterns observed must be considered as only preliminary because of the study design limitations and confounding factors that called for cautious interpretation of the results. For example, the RS^3^ worms were collected for RNA extraction at 9 days post drug treatment, resulting in a temporal separation between the stage of drug effect (and selection) and the stage at which gene expression was measured. In addition, potential sex-ratio heterogeneity among samples (that consisted of 300–500 mixed-sex adult worms) while expected to be close to 50/50 was not fully controlled for, which could result in expression variation in gender associated genes.

### Variant analysis and introgression mapping by whole-genome sequencing of population pools

Genomic DNA samples from pooled individuals of 300–500 RS^3^ or S_inbred_ worms were subjected to Illumina GAII, GAIIx, HiSeq 2000, HiSeq 2500 and MiSeq paired-end sequencing (S16 Table). Sequencing adapters were removed using trimmomatic (v.0.33) [92], and the resulting reads were aligned to the S_inbred_ reference assembly of *T*. *circumcincta* using BWA-MEM (v.0.7.12) [93]. Picard (v.1.95) was used to remove duplicate reads and local re-alignments were performed around indels using GATK (v. 3.4–46) [94]. Variants were called from the RS^3^ and S_inbred_ population pools using GATK HaplotypeCaller (v. 3.4–46) [94]. In line with the developers’ recommendations for analyzing pooled DNA samples (as opposed to diploid individuals),—sample_ploidy parameter was set to 10. In addition to requiring a minimum mapping quality score of 20 and a minimum base quality score of 20 in the reference alignment, the following set of quality filters were applied to SNP calls using GATK VariantFiltration (v. 3.4–46) [94]: DP (maximum depth) > median depth+(median absolute deviation×1.4826)×3; QD (variant confidence divided by the unfiltered depth of non-reference samples) < 2.0; FS (Phred-scaled p-value using Fisher’s Exact Test to detect strand bias in the reads) > 60.0; MQ (Root Mean Square of the mapping quality of the reads across all samples) < 40.0; MQRankSum (Mann-Whitney Rank Sum Test for mapping qualities) < -12.5; ReadPosRankSum (Mann-Whitney Rank Sum Test for the distance from the end of the read for reads with the alternative allele) < -8.0. Population allele frequency was estimated based on the relative abundance of reads supporting each allele (i.e., allelic depth), and Fisher’s exact test was used to assess the statistical significance of allele frequency differences between the populations. To delineate the introgressed loci in the RS^3^ strain, we conducted a genome-wide scan of fixation index (*F*_ST_) using nucleotide frequencies at polymorphic sites, and identified genomic regions that were most divergent relative to the parental S_inbred_ genetic background. Our mapping approach was based on the expectation that, after serial backcrossing and drug screening, causal and closely linked SNPs in the RS^3^ strain would retain the allelic profile of the anthelmintic resistant parental R_par_ isolate, whereas the genomic regions not associated with drug resistance would be represented by either the S_inbred_ genotype or a mixture of the two parental genotypes (depending on the variation in the recombination breakpoints among individuals). A SNP site was included in the *F*_ST_ analysis if it was supported by at least two alternative reads, did not overlap with indels, and had a minimum depth of 25× coverage in both populations. Following methods described by Kofler et al. [95] and assuming a pool size of 300 individuals in each population, *F*_ST_ values were estimated per site and averaged over non-overlapping 1-kb sliding windows. In addition, the number of loci meeting the depth of coverage threshold (25×) was examined for each window, and those windows with covered fraction > 0.5 (total combined length = 325.7 Mb) were included in the analysis. Position-sorted mean *F*_ST_ values (for each 1-kb window) were scanned for peaks after applying a kernel smoothing algorithm with adaptive bandwidth selection using the lokern package in R [96, 97] to identify blocks of genomic regions with extended linkage disequilibrium and elevated *F*_ST_, while reducing the effects of sequencing error, mapping artifacts, and base-to-base variation in coverage. Outlier windows were identified based on the empirical distribution of the smoothed *F*_ST_ values. A z-score of 4.5 was chosen as the cutoff threshold, guided by the z-score exhibited by the β-tubulin gene (TELCIR_01271; z-score = 4.66), a widely recognized BZ-resistance conferring locus in trichostrongylid nematodes. We reasoned that β-tubulin isotype 1 could effectively serve as a “positive control” and that loci with *F*_ST_ values similar to or more extreme than that for β-tubulin gene represented candidate loci worthy of further investigation. Statistical enrichment of GO terms among the genes overlapping outlier regions was assessed using a conditional hypergeometric test implemented in GOstat [98]. Gene-wise *F*_ST_ values per protein-coding loci were calculated as the maximum smoothed *F*_ST_ value among 1-kb windows that overlap the gene footprint (exon + intron). Using SnpEff (v.3.5), variants were annotated on the basis of their genomic location (e.g., exon, intron, intergenic, upstream/downstream (5 kb flanking regions) or splice site donor/acceptor), and their mutational effects were predicted (e.g., missense, nonsense or silent). Segregating haplotypes were reconstructed using either a phasing approach supported by sequencing reads [99, 100], or a *de novo* assembly-based method [101]. DNA copy number variation (CNV) was examined using CNV-seq [102] with p-value parameter set to 0.00001. Two-dimensional allele frequency spectra for RS^3^ and S_inbred_ populations were produced after alleles were subsampled without replacement to a uniform coverage of 10×. Only bi-allelic sites with a minimum coverage of 10× in both populations were included in the analysis, while SNPs with high depth of coverage (within the top 5% of the empirical distribution) were excluded. In total, 14,562,483 SNPs were used to obtain frequency spectra. Based on these variants, Tajima's D statistic was computed to assess whether allele frequency distributions deviated from neutral expectations [103].

### F2 mapping of IVM resistance loci using single worm double-digest restriction-site associated DNA sequencing (ddRAD-seq)

A segregating F2 mapping population of *T*. *circumcincta* was generated as part of the work undertaken to introgress anthelmintic resistance genes into an anthelmintic susceptible genetic background. A cross was initiated by surgically transferring ~300 anthelmintic resistant adult males (R_par_) and an equivalent number of susceptible late L4 stage females (S_inbred_) into the abomasum of a previously worm-free kid goat. F1 eggs collected from the host feces were then cultured and ~10,000 of the resulting infective larvae were used to infect a second worm-free kid goat to produce an F_2_ generation. Two experiments were carried out to investigate the segregation of resistance to each class of anthelmintic, one using kid goats as the recipients and a second using lambs, with essentially the same results. We describe the lamb experiment here (S14 Table). Two groups of worm-free lambs (n = 27 per group) were administered an oral dose of ~8,000 (Group 1) or 16,000 (Group 2) of the F_2_ generation infective larvae each. On Day 27 post-infection, the lambs in Group 2 were treated with IVM. Those in Group 1 remained untreated. Three days later (Day 30 post-infection) individual fecal nematode egg counts (FECs) were undertaken on all animals and, using a restricted randomization procedure based on weight and FEC, each of the infection groups was subdivided into 3 equal-sized anthelmintic treatment groups (1a-1c and 2a-2c). On Day 31 post-infection, anthelmintic treatments were administered as follows: Groups 1b and 2b received BZ while Groups 1c and 2c received LEV. Groups 1a and 2a remained untreated as controls. Anthelmintic doses were calculated on the basis of individual live-weights and each dose was administered orally with a disposable syringe. For ddRAD-seq analysis, a total of 24 adult male F_2_ IVM-treatment survivors from the parallel, kid goat experiment (Group 2a) and 24 drug-naïve adult male (Group 1a) F_2_ worms also from the kid goat experiment were individually transferred into 100 μl of DirectPCR Lysis Reagent (Mouse Tail; Viagen Biotech, Los Angeles, CA), supplemented with 3% proteinase K (10 mg/ml; Roche) and incubated at 55°C for 16 h followed by 90°C for 1 h to denature the proteinase K. A 20 μl volume from each worm lysate (~10 ng DNA) was digested with EcoRI and MspI overnight, after which sequencing adapters (P1-EcoRI-inline-barcode and P2-MspI) were ligated to the fragment termini. The reaction was purified using a 0.5X and 0.7X double size selection (modified from Lennon et al. [104]) using Agencourt AmpureXP beads (Beckman Coulter, Brea, CA), and PCR amplified to incorporate index sequences for multiplexing using KAPA HiFi Real Time master mix using the following protocol; 98°C for 2 min, followed by 14 cycles of 98°C for 15 sec, 60°C for 30 sec and 72°C for 30 sec. PCR reactions were purified using AmpureXP beads, after which the DNA concentrations were standardized using a Qubit 3.0 Fluorometer (Life Technologies, Carlsbad, CA) and samples pooled at equimolar concentration. Adapter-ligated and PCR amplified fragments approximately 500-600bp in length were obtained by gel size selection and purification. The ddRADseq library, supplemented with a 10% PhiX spike-in control, was sequenced using an Illumina MiSeq (reagent kit v3), resulting in 150bp single-end sequencing reads. Sequencing data were demultiplexed using process_radtags [105], and were mapped to the *T*. *circumcincta* reference assembly using BWA-MEM (v0.7.10) [93]. Local realignments were performed around indels using the GATK (v3.3–0), after which variants were called by HaplotypeCaller under default parameters. *F*_ST_ estimation [106] was carried out using VCFtools (v.0.1.12b) [107].

### Genotyping β-tubulin alleles

An allele-specific multiplex PCR strategy based on that developed by Humbert and Elard [108] was used in a 96-reaction format to assess the presence of a Phe (TTC)/Tyr (TAC) substitution at codon 200 in the β-tubulin *isotype-1* gene in individual worms from the anthelmintic susceptible (S_inbred_) and multiple-anthelmintic resistant (RS^3^) strains of *T*. *circumcincta*. Only adult male worms were used for these allele-specific reactions to avoid the possibility of DNA from sperm and/or fertilised eggs present in female worms interfering with the genotype identifications. The strategy involved the use of four primers per reaction, two of which–one forward and one reverse–were generic (allele-nonspecific), while the remaining two–again one forward and one reverse–were allele-specific (S2 Fig). Primer designs, which differed slightly from those of Humbert and Elard [108] to account for minor DNA sequence differences between the strains studied by them and those used in the present study, were as follows: generic forward [“TubGF”] 5′ CTTAGATGTTGTTCGTAAAGAGG 3′; generic reverse [“TubGR”] 5′ CATGTTCACAGCCAACTTGC 3′; Phe-specific [“TubSASRev”] 5′ AGAGCTTCATTATCGATGCAGA 3′; Tyr-specific [“TubRASFwd”] 5′ TGGTWGAAAAYACCGATGAAACRTA 3′. Note that TubRASFwd was degenerate at three nucleotide positions (5, 11 and 23) in order to accommodate the presence of SNPs in those positions in some haplotypes containing a Tyr at codon 200 (*see* S3 Fig). Two further primers–[“TubRASH3Rev”] [5′ CTTCATTATCGATGCAGAATGTTAA 3′] and [“TubSASH1Fwd”] [5′ CAGTTGGTTGAAAATACCGATGA 3′]–were designed to detect the presence or absence of a Glu198Leu substitution.

### Real-time PCR quantification of single worm *Tci-pgp-9* copy number

Adult worms from each of the above *T*. *circumcincta* strains were isolated from experimentally infected goats (approximately one week after successive treatments with all three anthelmintic families in the case of RS_3_), washed free of all debris in physiological saline and then transferred, in batches of either 100 male worms or ~300 mixed-sex worms, into cryovials where they were frozen in liquid nitrogen until required. Crude genomic DNA template was prepared from individual adult male *T*. *circumcincta* from the S_inbred_ and RS_3_ strains (96/strain) by overnight incubation in lysis solution [Viagen DirectPCR (MouseTail), 50 μl per worm with 3 mg/ml ProteinaseK] without further purification. SYBR^®^Green real-time PCR assays were performed in a GeneAmp 5700 sequence detection system to compare *Tci-pgp-9* gene copy number in individual male worms from the S_inbred_ and RS_3_ worm populations. Primers constructed for this purpose corresponded to genomic DNA sequence within the first putative inter-nucleotide binding domain of *Tci-pgp-9* (i.e., *Tci-pgp-9-IBDA*) and were designed to amplify an equivalent 99bp product from each of the seven *Tci-pgp-9-IBDA* haplotypes identified from the S_inbred_ and/or RS^3^ strain worms. Primer sequences were as follows: forward [“IBD77RTGF”] 5′ CGHTATGGACGTGAAAAAGTCACAGA 3′ and reverse [“IBD77RTGR”] 5′ CCAACTCACGTCRGGGAAYGACTG 3′. Although designs of both these primers were based on well conserved *Tci-pgp-9* IBDA haplotypes (*see* S6 Fig) it was necessary to incorporate some degeneracy to ensure perfect matches in all cases. To take variation in the concentration of genomic DNA between single worm DNA preparations, *Tci-pgp-9* copy number was calculated in reference to a single copy gene. The single copy reference in this case was *T*. *circumcincta* β-tubulin *isotype-1* using primers forward [“TUBRTGF2”] 5′ GGGCTTCCAACTGACGCATTCTTTG 3′ and reverse [“TUBRTGR2’] 5′ GGGCTTCCAACTGACGCATTCTTTG 3′ which amplified a 122bp product from an exon in the central region of the gene. The *Tm* for both primers was within the same range as those of IBD77RTGF and IBD77RTGR. All reactions were performed in duplicate in 96-well optical reaction plates (Applied Biosystems) using 25 μl reaction volumes which contained SYBR^®^Green PCR mastermix (Applied Biosystems), 0.2 μM of each gene-specific primer and 1 μl of 10-fold diluted crude genomic template. For both the target and reference genes “no-template controls” were included on each plate. Amplification conditions for the above reactions were as follows: initial incubation at 50°C for 2 min, followed by 95°C for 10 min to denature the template, followed by 40 cycles of 95°C for 15 sec and 60°C for 1 min. Following the reactions a melting curve and cycle threshold (*C*_*T*_) value were generated for each sample. The *C*_*T*_ value indicates the fractional cycle number at which the amount of amplified DNA reaches a fixed threshold. Mean *C*_*T*_ values of duplicate samples were used in subsequent quantification analyses. No product was amplified in the “no-template control” reactions. As indicated above the amount of target measured in each case was standardised in relation to an endogenous reference gene to account for any between-worm variation in the total amount of gDNA template available. This was done by calculating *ΔC*_*T*_ values for each sample–the *ΔC*_*T*_ value indicates the difference in cycle number required to reach the fixed threshold for the target and reference genes. Two-sample *t*-tests were used to compare *ΔC*_*T*_ values for worms from the S_inbred_ and RS^3^ populations, as well as specific genotype groups within the RS^3^ population.

### Genotyping of individual S_inbred_ and RS^3^ worms using allele-specific PCR

*Tci-pgp-9-IBDA* haplotypes were identified based on PCR clones (n = 66) amplified from gDNA preparations (see S6 Fig). “Allele-specific” primers were designed to differentiate between each of the haplotypes using a nested PCR strategy to allow genotyping of individual male worms from the S_inbred_ and RS^3^ strains of *T*. *circumcincta*. Primary reactions were performed using the degenerate primers based on the following amino acid sequences: VEIDKINIE (sense) [“IBD77GF3”] and GTQMSGGQ (antisense) [“IBD77GR2”]. Reactions were carried out in a Mastercycler thermal cycler (96 well block) using 20 μl reaction volumes containing 0.5 unit Platinum *Taq* polymerase (Invitrogen), 2 μl 10x *Taq* buffer, 2.5 mM MgCl_2_, 200 μM each dNTP, 20 pmol of each primer and 2 μl template. A touchdown protocol was used as follows: 95°C for 8 min to denature the template and activate the enzyme, followed by 12 cycles of 94°C for 30 sec, 58°C (–0.5°C/cycle) for 30 sec and 72°C for 1 min, followed by 28 cycles of 94°C for 30 sec, 52°C for 30 sec and 72°C for 1 min, and finishing with a final elongation step of 72°C for 7 min. A single generic (“allele-nonspecific”) forward primer–“IBD77GF5” [5′ GAGTAGTKTCACARGARCCNATGCT 3′]–was used for all subsequent nested allele-specific reactions. This primer was degenerate at four nucleotide positions (8, 14, 17 and 20) to accommodate the presence of SNPs at those positions in some haplotypes. The allele-specific reverse primers used in combination with IBD77GF5 to assess worm genotypes are shown in S17 Table. Allele-specific reactions were similarly performed in a Mastercycler using 20μl reaction volumes but unlike the first round reactions they contained 1.0 mM MgCl_2_ and 10 pmol of each primer, and amplification conditions used were more stringent than for the first round reactions, i.e., 95°C for 8 min, followed by 35 cycles of 94°C for 30 sec, 60–61°C for 30 sec and 72°C for 1 min, finishing with a final elongation step of 72°C for 7 min. Five microliters of each reaction were run on a 2% agarose gel in the presence of ethidium bromide to assess the incidence of each of the sequence variants in each of the worm populations. *Tci-pgp-9- IBDA* genotype information derived from the allele-specific reactions was checked and verified in selected worms from each population by sequencing PCR fragments amplified from these worms using a nested protocol similar to that used for the allele-specific reactions.

### Comparison of cDNA sequences encoding the N-terminal and C-terminal transmembrane regions of Tci-PGP-9 protein molecules from S_inbred_ and RS^3^ strain worms

Total RNA was isolated from mixed-sex batches of adult worms from each of the S_inbred_ and RS^3^ strains using TRI REAGENT LS (Molecular Research Center, Cincinnati, OH). Synthesis of first-strand complementary DNA (cDNA) was carried out using SuperScript II Reverse Transcriptase (Invitrogen) and poly(A) oligo(dT)_12-18_ primer (Invitrogen) as per the manufacturer’s instructions. The resulting cDNA solution was diluted with DEPC-treated water to equate to an initial RNA concentration of 20 ng/μl before being stored at -20°C until required for subsequent PCR. Overlapping fragments, encoding the complete transmembrane region from each half of the *T*. *circumcincta* PGP-9 protein molecule, were amplified from first-strand cDNA derived from S_inbred_ (two separate pools) and RS^3^ worms using degenerate primers in nested or partially nested PCRs. Primer designs were based on the deduced amino acid sequence of Tci-PGP-9, and corresponded to: N-terminal transmembrane region, fragment 1, first round reactions–DAILVCFQ (sense) [“PGP9AF”]/ MIICGAFI (antisense) [“PGP9AR”]; nested reactions–VCFQFRYT (sense) [“PGP9AFnest”]/ APFMIICG (antisense) [“PGP9ARnest”]; N-terminal transmembrane region, fragment 2, first round reactions–GGFIVAFT (sense) [“PGP9BF”]/ YNPADGKI (antisense) [“PGP9BR”]; nested reactions–IVAFTYDW (sense) [“PGP9BFnest”]/ GCGKSTII (antisense) [“PGP9BRnest”]; C-terminal transmembrane region, fragment 1, first round reactions–VTEDTGVA (sense) [“PGP9CF”]/ QAIQMKFM (antisense) [“PGP9CR”]; nested reactions–ATAQNDP (sense) [“PGP9CFnest”]/ PGP9CR; C-terminal transmembrane region, fragment 2, first round reactions–IALYFGW (sense) [“PGP9DF”]/ GCGKSTVI (antisense) [“PGP9DR”]; nested reactions–LYFGWQMA (sense) [“PGP9DFnest”]/ VGPSGCG (antisense) [“PGP9DRnest”]. Approximate locations of these primer sites in relation to each other and to the expected transmembrane structures and the ATP sites of the PGP-9 protein molecule are depicted in S7 Fig. Although appropriate products were amplified in each case using the above primer combinations, it subsequently became apparent that there were errors in the design of the sense primers PGP9AF and PGP9AFnest. These in fact should have corresponded to VPKASIGQ and IGQLFRYT respectively as indicated in S9 Fig. All PCR amplifications were performed in an MJ Research PTC-200 thermal cycler using final volumes of 20 μl containing 0.5 unit Platinum *Taq* polymerase (Invitrogen), 2 μl *Taq* buffer, 2.5 mM MgCl_2_, 200 μM each dNTP, 20 pmol of each primer and 2 μl cDNA template. Both first round and nested reactions were undertaken using a touchdown PCR procedure as follows: 95°C for 5 min to denature the template, followed by 12 cycles of 94°C for 15 sec, 58°C (–0.5°C/cycle) for 30 sec and 72°C for 1 min, followed by 28 cycles of 94°C for 15 sec, 52°C for 30 sec and 72°C for 1 min, and finishing with a final elongation step of 72°C for 7 min. PCR products (*see* S8 Fig**)** were ligated into a TOPO TA Cloning vector (Invitrogen) and multiple clones sequenced for each product.

## Supporting information

S1 Fig Reconstructed haplotypes of β-tubulin *isotype-1* in *Teladorsagia circumcincta* RS3 population. (PDF)

S2 Fig Allele-specific multiplex PCR strategy to assess the presence of a F200Y (tTc/tAc) substitution in the β-tubulin *isotype-1* gene in individual male *Teladorsagia circumcincta*. (PDF)

S3 Fig ClustalW multiple alignment of selected gDNA sequence variants from the central region of the β-tubulin *isotype-1* gene from S_inbred_ and RS^3^ worms showing amino acid codon positions 167, 198 and 200, SNPs and positions of introns. (PDF)

S4 Fig Prediction of signal peptide cleavage site in Tci-LGC-54 (TELCIR_00170). (PDF)

S5 Fig Distribution of *ΔC*_*T*_ values of individual male worms from the *T*. *circumcincta* strains RS^3^ and S_inbred_. (PDF)

S6 Fig Multiple-alignment of partial sequences of *Tci-pgp-9*-IBDA haplotypes showing introns, amino acid translation and locations of allele-specific reverse primers used in genotyping reactions. (PDF)

S7 Fig Approximate locations of the oligonucleotide primer sites used to amplify cDNA fragments encoding the N-terminal and C-terminal transmembrane regions of Tci-PGP-9 protein molecules from worms from the S_inbred_ and RS^3^ strains. (PDF)

S8 Fig Agarose gel showing cDNA products amplified by PCR from the N-terminal transmembrane region of *Tci-pgp-9*. (PDF)

S9 Fig Deduced amino acid sequence of Tci-PGP-9 showing relative positions of amino acid substitutions associated with the multiple-anthelmintic resistant RS^3^ strain of *Teladorsagia circumcincta* relative to the anthelmintic susceptible S_inbred_ counterpart. (PDF)

S10 Fig Fixation index (*F*_ST_) of ddRAD-seq derived SNP markers between IVM-screened and drug-naïve F2 mapping populations of *Teladorsagia circumcincta*. (PDF)

S1 Table Summary of *Teladorsagia circumcincta* genomic features and comparison to other clade V nematodes. (PDF)

S2 Table Amino acid composition (%). (PDF)

S3 Table *Teladorsagia circumcincta* repeat library characterization. (XLSX)

S4 Table Length of the *Teladorsagia circumcincta* genome and coding sequences (CDS) that are considered “callable” based on mapping coverage and quality. (PDF)

S5 Table Number of fixed and segregating bi-allelic SNPs in *Teladorsagia circumcincta* S_inbred_ and RS^3^ strains. (PDF)

S6 Table Outlier regions (z-score > 4.5) identified through a genome-wide scan of fixation index (*F*_ST_) between S_inbred_ and RS^3^ populations of *Teladorsagia circumcincta*. (XLSX)

S7 Table Fixation index (*F*_ST_) values, copy number variation (CNV) ratios, and RNA-seq transcript abundance ratios per gene. (XLSX)

S8 Table Nonsynonymous variants in *F*_ST_-outlier candidate genes. (XLSX)

S9 Table Prevalence of β-tubulin genotypes in inbred anthelmintic susceptible (S_inbred_) and multiple-anthelmintic resistant (RS^3^) populations of *Teladorsagia circumcincta*. (PDF)

S10 Table Summary of *ΔC*_*T*_ values in relation to the occurrence of *Tci-pgp-9-IBDA* haplotypes in male worms of the RS^3^ strain of *Teladorsagia circumcincta*. (PDF)

S11 Table Fixation index (*F*_ST_) of ddRAD-seq derived SNP markers between IVM-screened and drug-naïve F2 mapping populations of *Teladorsagia circumcincta*. (XLSX)

S12 Table Genes displaying a minimum *F*_ST_ z-score of 2.5 in both the introgression and the F2 mapping experiments. (PDF)

S13 Table IVM selection signals in the introgression *F*_ST_ outlier genes. (XLSX)

S14 Table Summary of experimental design showing lamb treatment groups, *T*. *circumcincta* infecting doses, final group sizes (*N*), ivermectin treatment status and 2° anthelmintic treatments applied. (PDF)

S15 Table Between-infection-group comparisons of post-mortem worm burdens, following oxfendazole or levamisole treatment, in host animals infected with ivermectin-screened or unscreened R_par_ x S_inbred_ F_2_ generation *Teladorsagia circumcincta*. (PDF)

S16 Table Summary of sequenced gDNA and mRNA libraries. (PDF)

S17 Table Allele-specific reverse PCR primers used in *Tci-pgp-9-IBDA* genotyping reactions. (PDF)

## References

[1] Geary TG. Are new anthelmintics needed to eliminate human helminthiases? Current opinion in infectious diseases. 2012;25(6):709–17. doi: 10.1097/QCO.0b013e328359f04a 23041774 10.1097/QCO.0b013e328359f04a

[2] Kaplan RM. Drug resistance in nematodes of veterinary importance: a status report. Trends in parasitology. 2004;20(10):477–81. doi: 10.1016/j.pt.2004.08.001 15363441 10.1016/j.pt.2004.08.001

[3] Prichard RK, Basanez MG, Boatin BA, McCarthy JS, Garcia HH, Yang GJ, et al A research agenda for helminth diseases of humans: intervention for control and elimination. PLoS neglected tropical diseases. 2012;6(4):e1549 doi: 10.1371/journal.pntd.0001549 22545163 10.1371/journal.pntd.0001549 PMC3335868

[4] Gilleard JS, Beech RN. Population genetics of anthelmintic resistance in parasitic nematodes. Parasitology. 2007;134(Pt 8):1133–47. doi: 10.1017/S0031182007000066 17608973 10.1017/S0031182007000066

[5] James CE, Hudson AL, Davey MW. Drug resistance mechanisms in helminths: is it survival of the fittest? Trends in parasitology. 2009;25(7):328–35. doi: 10.1016/j.pt.2009.04.004 19541539 10.1016/j.pt.2009.04.004

[6] Gilleard JS. Understanding anthelmintic resistance: the need for genomics and genetics. Int J Parasitol. 2006;36(12):1227–39. doi: 10.1016/j.ijpara.2006.06.010 16889782 10.1016/j.ijpara.2006.06.010

[7] Kotze AC, Hunt PW, Skuce P, von Samson-Himmelstjerna G, Martin RJ, Sager H, et al Recent advances in candidate-gene and whole-genome approaches to the discovery of anthelmintic resistance markers and the description of drug/receptor interactions. Int J Parasitol Drugs Drug Resist. 2014;4(3):164–84. doi: 10.1016/j.ijpddr.2014.07.007 25516826 10.1016/j.ijpddr.2014.07.007 PMC4266812

[8] Gilleard JS. Haemonchus contortus as a paradigm and model to study anthelmintic drug resistance. Parasitology. 2013;140(12):1506–22. doi: 10.1017/S0031182013001145 23998513 10.1017/S0031182013001145

[9] Braisher TL, Gemmell NJ, Grenfell BT, Amos W. Host isolation and patterns of genetic variability in three populations of Teladorsagia from sheep. Int J Parasitol. 2004;34(10):1197–204. doi: 10.1016/j.ijpara.2004.06.005 15380691 10.1016/j.ijpara.2004.06.005

[10] Anderson TJ, Blouin MS, Beech RN. Population biology of parasitic nematodes: applications of genetic markers. Advances in parasitology. 1998;41:219–83. 9734295 10.1016/s0065-308x(08)60425-x

[11] Blouin MS, Yowell CA, Courtney CH, Dame JB. Host movement and the genetic structure of populations of parasitic nematodes. Genetics. 1995;141(3):1007–14. 8582607 10.1093/genetics/141.3.1007 PMC1206824

[12] Redman E, Sargison N, Whitelaw F, Jackson F, Morrison A, Bartley DJ, et al Introgression of ivermectin resistance genes into a susceptible Haemonchus contortus strain by multiple backcrossing. PLoS pathogens. 2012;8(2):e1002534 doi: 10.1371/journal.ppat.1002534 22359506 10.1371/journal.ppat.1002534 PMC3280990

[13] Le Jambre LF, Lenane IJ, Wardrop AJ. A hybridisation technique to identify anthelmintic resistance genes in Haemonchus. Int J Parasitol. 1999;29(12):1979–85. 10961854 10.1016/s0020-7519(99)00157-5

[14] Chevalier FD, Valentim CL, LoVerde PT, Anderson TJ. Efficient linkage mapping using exome capture and extreme QTL in schistosome parasites. BMC Genomics. 2014;15:617 doi: 10.1186/1471-2164-15-617 25048426 10.1186/1471-2164-15-617 PMC4117968

[15] Bisset SA. The genetic basis of multiple-anthelmintic resistance in *Teladorsagia circumcincta*, a gastrointestinal nematode parasite of sheep and goats. PhD thesis. Flinders University of South Australia. 2007.

[16] Pomroy WE. Anthelmintic resistance in New Zealand: a perspective on recent findings and options for the future. New Zealand veterinary journal. 2006;54(6):265–70. doi: 10.1080/00480169.2006.36709 17151723 10.1080/00480169.2006.36709

[17] Scott I, Pomroy WE, Kenyon PR, Smith G, Adlington B, Moss A. Lack of efficacy of monepantel against Teladorsagia circumcincta and Trichostrongylus colubriformis. Vet Parasitol. 2013;198(1–2):166–71. doi: 10.1016/j.vetpar.2013.07.037 23953148 10.1016/j.vetpar.2013.07.037

[18] Parra G, Bradnam K, Korf I. CEGMA: a pipeline to accurately annotate core genes in eukaryotic genomes. Bioinformatics. 2007;23(9):1061–7. doi: 10.1093/bioinformatics/btm071 17332020 10.1093/bioinformatics/btm071

[19] Barriere A, Yang SP, Pekarek E, Thomas CG, Haag ES, Ruvinsky I. Detecting heterozygosity in shotgun genome assemblies: Lessons from obligately outcrossing nematodes. Genome Res. 2009;19(3):470–80. doi: 10.1101/gr.081851.108 19204328 10.1101/gr.081851.108 PMC2661809

[20] Laing R, Kikuchi T, Martinelli A, Tsai IJ, Beech RN, Redman E, et al The genome and transcriptome of Haemonchus contortus, a key model parasite for drug and vaccine discovery. Genome Biol. 2013;14(8):R88 doi: 10.1186/gb-2013-14-8-r88 23985316 10.1186/gb-2013-14-8-r88 PMC4054779

[21] Huang S, Chen Z, Huang G, Yu T, Yang P, Li J, et al HaploMerger: reconstructing allelic relationships for polymorphic diploid genome assemblies. Genome Res. 2012;22(8):1581–8. doi: 10.1101/gr.133652.111 22555592 10.1101/gr.133652.111 PMC3409271

[22] Driscoll M, Dean E, Reilly E, Bergholz E, Chalfie M. Genetic and molecular analysis of a Caenorhabditis elegans beta-tubulin that conveys benzimidazole sensitivity. The Journal of cell biology. 1989;109(6 Pt 1):2993–3003. 2592410 10.1083/jcb.109.6.2993 PMC2115974

[23] Saunders GI, Wasmuth JD, Beech R, Laing R, Hunt M, Naghra H, et al Characterization and comparative analysis of the complete Haemonchus contortus beta-tubulin gene family and implications for benzimidazole resistance in strongylid nematodes. Int J Parasitol. 2013;43(6):465–75. doi: 10.1016/j.ijpara.2012.12.011 23416426 10.1016/j.ijpara.2012.12.011

[24] Von Samson-Himmelstjerna G, Blackhall WJ, McCarthy JS, Skuce PJ. Single nucleotide polymorphism (SNP) markers for benzimidazole resistance in veterinary nematodes. Parasitology. 2007;134(Pt 8):1077–86. doi: 10.1017/S0031182007000054 17608967 10.1017/S0031182007000054

[25] Beech RN, Prichard RK, Scott ME. Genetic variability of the beta-tubulin genes in benzimidazole-susceptible and -resistant strains of Haemonchus contortus. Genetics. 1994;138(1):103–10. 8001777 10.1093/genetics/138.1.103 PMC1206121

[26] Kwa MS, Kooyman FN, Boersema JH, Roos MH. Effect of selection for benzimidazole resistance in Haemonchus contortus on beta-tubulin isotype 1 and isotype 2 genes. Biochem Biophys Res Commun. 1993;191(2):413–9. doi: 10.1006/bbrc.1993.1233 8096381 10.1006/bbrc.1993.1233

[27] Kwa MS, Veenstra JG, Van Dijk M, Roos MH. Beta-tubulin genes from the parasitic nematode Haemonchus contortus modulate drug resistance in Caenorhabditis elegans. Journal of molecular biology. 1995;246(4):500–10. doi: 10.1006/jmbi.1994.0102 7877171 10.1006/jmbi.1994.0102

[28] Ghisi M, Kaminsky R, Maser P. Phenotyping and genotyping of Haemonchus contortus isolates reveals a new putative candidate mutation for benzimidazole resistance in nematodes. Vet Parasitol. 2007;144(3–4):313–20. doi: 10.1016/j.vetpar.2006.10.003 17101226 10.1016/j.vetpar.2006.10.003

[29] Redman E, Whitelaw F, Tait A, Burgess C, Bartley Y, Skuce PJ, et al The emergence of resistance to the benzimidazole anthlemintics in parasitic nematodes of livestock is characterised by multiple independent hard and soft selective sweeps. PLoS neglected tropical diseases. 2015;9(2):e0003494 doi: 10.1371/journal.pntd.0003494 25658086 10.1371/journal.pntd.0003494 PMC4319741

[30] Aguayo-Ortiz R, Mendez-Lucio O, Romo-Mancillas A, Castillo R, Yepez-Mulia L, Medina-Franco JL, et al Molecular basis for benzimidazole resistance from a novel beta-tubulin binding site model. J Mol Graph Model. 2013;45:26–37. doi: 10.1016/j.jmgm.2013.07.008 23995453 10.1016/j.jmgm.2013.07.008

[31] Devillers-Thiery A, Galzi JL, Eisele JL, Bertrand S, Bertrand D, Changeux JP. Functional architecture of the nicotinic acetylcholine receptor: a prototype of ligand-gated ion channels. J Membr Biol. 1993;136(2):97–112. 7508983 10.1007/BF02505755

[32] Jones AK, Davis P, Hodgkin J, Sattelle DB. The nicotinic acetylcholine receptor gene family of the nematode Caenorhabditis elegans: an update on nomenclature. Invert Neurosci. 2007;7(2):129–31. doi: 10.1007/s10158-007-0049-z 17503100 10.1007/s10158-007-0049-z PMC2972647

[33] Holden-Dye L, Joyner M, O'Connor V, Walker RJ. Nicotinic acetylcholine receptors: a comparison of the nAChRs of Caenorhabditis elegans and parasitic nematodes. Parasitology international. 2013;62(6):606–15. doi: 10.1016/j.parint.2013.03.004 23500392 10.1016/j.parint.2013.03.004

[34] Neveu C, Charvet CL, Fauvin A, Cortet J, Beech RN, Cabaret J. Genetic diversity of levamisole receptor subunits in parasitic nematode species and abbreviated transcripts associated with resistance. Pharmacogenet Genomics. 2010;20(7):414–25. doi: 10.1097/FPC.0b013e328338ac8c 20531256 10.1097/FPC.0b013e328338ac8c

[35] Boulin T, Fauvin A, Charvet CL, Cortet J, Cabaret J, Bessereau JL, et al Functional reconstitution of Haemonchus contortus acetylcholine receptors in Xenopus oocytes provides mechanistic insights into levamisole resistance. Br J Pharmacol. 2011;164(5):1421–32. doi: 10.1111/j.1476-5381.2011.01420.x 21486278 10.1111/j.1476-5381.2011.01420.x PMC3221097

[36] Buxton SK, Charvet CL, Neveu C, Cabaret J, Cortet J, Peineau N, et al Investigation of acetylcholine receptor diversity in a nematode parasite leads to characterization of tribendimidine- and derquantel-sensitive nAChRs. PLoS pathogens. 2014;10(1):e1003870 doi: 10.1371/journal.ppat.1003870 24497826 10.1371/journal.ppat.1003870 PMC3907359

[37] Betts MJ, Russell RB. Amino Acid Properties and Consequences of Substitutions In: Barnes MR, Gray IC, editors. Bioinformatics for Geneticists. Hoboken: John Wiley & Sons; 2003 p. 289–316.

[38] Williamson SM, Storey B, Howell S, Harper KM, Kaplan RM, Wolstenholme AJ. Candidate anthelmintic resistance-associated gene expression and sequence polymorphisms in a triple-resistant field isolate of Haemonchus contortus. Mol Biochem Parasitol. 2011;180(2):99–105. doi: 10.1016/j.molbiopara.2011.09.003 21945142 10.1016/j.molbiopara.2011.09.003

[39] Sarai RS, Kopp SR, Coleman GT, Kotze AC. Acetylcholine receptor subunit and P-glycoprotein transcription patterns in levamisole-susceptible and -resistant Haemonchus contortus. Int J Parasitol Drugs Drug Resist. 2013;3:51–8. doi: 10.1016/j.ijpddr.2013.01.002 24533293 10.1016/j.ijpddr.2013.01.002 PMC3862433

[40] Sarai RS, Kopp SR, Coleman GT, Kotze AC. Drug-efflux and target-site gene expression patterns in Haemonchus contortus larvae able to survive increasing concentrations of levamisole in vitro. Int J Parasitol Drugs Drug Resist. 2014;4(2):77–84. doi: 10.1016/j.ijpddr.2014.02.001 25057457 10.1016/j.ijpddr.2014.02.001 PMC4095050

[41] Kagawa H, Takuwa K, Sakube Y. Mutations and expressions of the tropomyosin gene and the troponin C gene of Caenorhabditis elegans. Cell Struct Funct. 1997;22(1):213–8. 9113409 10.1247/csf.22.213

[42] Gottschalk A, Almedom RB, Schedletzky T, Anderson SD, Yates JR 3rd, Schafer WR. Identification and characterization of novel nicotinic receptor-associated proteins in Caenorhabditis elegans. EMBO J. 2005;24(14):2566–78. doi: 10.1038/sj.emboj.7600741 15990870 10.1038/sj.emboj.7600741 PMC1176467

[43] Hobert O. The neuronal genome of *Caenorhabditis elegans* In: The *C. elegans* Research Community, editor. WormBook.2013. 10.1895/wormbook.1.161.1 PMC4781646 24081909

[44] Harris TW, Antoshechkin I, Bieri T, Blasiar D, Chan J, Chen WJ, et al WormBase: a comprehensive resource for nematode research. Nucleic Acids Res. 2010;38(Database issue):D463–7. doi: 10.1093/nar/gkp952 19910365 10.1093/nar/gkp952 PMC2808986

[45] Beech RN, Callanan MK, Rao VT, Dawe GB, Forrester SG. Characterization of cys-loop receptor genes involved in inhibitory amine neurotransmission in parasitic and free living nematodes. Parasitology international. 2013;62(6):599–605. doi: 10.1016/j.parint.2013.03.010 23602737 10.1016/j.parint.2013.03.010

[46] Ringstad N, Abe N, Horvitz HR. Ligand-gated chloride channels are receptors for biogenic amines in C. elegans. Science. 2009;325(5936):96–100. doi: 10.1126/science.1169243 19574391 10.1126/science.1169243 PMC2963310

[47] Pirri JK, McPherson AD, Donnelly JL, Francis MM, Alkema MJ. A tyramine-gated chloride channel coordinates distinct motor programs of a Caenorhabditis elegans escape response. Neuron. 2009;62(4):526–38. doi: 10.1016/j.neuron.2009.04.013 19477154 10.1016/j.neuron.2009.04.013 PMC2804440

[48] Rao VT, Siddiqui SZ, Prichard RK, Forrester SG. A dopamine-gated ion channel (HcGGR3*) from Haemonchus contortus is expressed in the cervical papillae and is associated with macrocyclic lactone resistance. Mol Biochem Parasitol. 2009;166(1):54–61. doi: 10.1016/j.molbiopara.2009.02.011 19428673 10.1016/j.molbiopara.2009.02.011

[49] Ghosh R, Andersen EC, Shapiro JA, Gerke JP, Kruglyak L. Natural variation in a chloride channel subunit confers avermectin resistance in C. elegans. Science. 2012;335(6068):574–8. doi: 10.1126/science.1214318 22301316 10.1126/science.1214318 PMC3273849

[50] Lynagh T, Lynch JW. Molecular mechanisms of Cys-loop ion channel receptor modulation by ivermectin. Front Mol Neurosci. 2012;5:60 doi: 10.3389/fnmol.2012.00060 22586367 10.3389/fnmol.2012.00060 PMC3345530

[51] Hibbs RE, Gouaux E. Principles of activation and permeation in an anion-selective Cys-loop receptor. Nature. 2011;474(7349):54–60. doi: 10.1038/nature10139 21572436 10.1038/nature10139 PMC3160419

[52] McCavera S, Walsh TK, Wolstenholme AJ. Nematode ligand-gated chloride channels: an appraisal of their involvement in macrocyclic lactone resistance and prospects for developing molecular markers. Parasitology. 2007;134(Pt 8):1111–21. doi: 10.1017/S0031182007000042 17608971 10.1017/S0031182007000042

[53] Wolstenholme AJ. Glutamate-gated chloride channels. J Biol Chem. 2012;287(48):40232–8. doi: 10.1074/jbc.R112.406280 23038250 10.1074/jbc.R112.406280 PMC3504739

[54] Urdaneta-Marquez L, Bae SH, Janukavicius P, Beech R, Dent J, Prichard R. A dyf-7 haplotype causes sensory neuron defects and is associated with macrocyclic lactone resistance worldwide in the nematode parasite Haemonchus contortus. Int J Parasitol. 2014;44(14):1063–71. doi: 10.1016/j.ijpara.2014.08.005 25224687 10.1016/j.ijpara.2014.08.005

[55] Guerrero J, Freeman AS. Amphids: the neuronal ultrastructure of macrocyclic-lactone-resistant Haemonchus contortus. Parassitologia. 2004;46(1–2):237–40. 15305725

[56] Dent JA, Smith MM, Vassilatis DK, Avery L. The genetics of ivermectin resistance in Caenorhabditis elegans. Proc Natl Acad Sci U S A. 2000;97(6):2674–9. 10716995 10.1073/pnas.97.6.2674 PMC15988

[57] Laing R, Maitland K, Lecova L, Skuce PJ, Tait A, Devaney E. Analysis of putative resistance gene loci in UK field populations of Haemonchus contortus after 6years of macrocyclic lactone use. Int J Parasitol. 2016;46(10):621–30. doi: 10.1016/j.ijpara.2016.03.010 27179994 10.1016/j.ijpara.2016.03.010 PMC5011429

[58] Rezansoff AM, Laing R, Gilleard JS. Evidence from two independent backcross experiments supports genetic linkage of microsatellite Hcms8a20, but not other candidate loci, to a major ivermectin resistance locus in Haemonchus contortus. Int J Parasitol. 2016;46(10):653–61. doi: 10.1016/j.ijpara.2016.04.007 27216082 10.1016/j.ijpara.2016.04.007

[59] Lespine A, Menez C, Bourguinat C, Prichard RK. P-glycoproteins and other multidrug resistance transporters in the pharmacology of anthelmintics: Prospects for reversing transport-dependent anthelmintic resistance. Int J Parasitol Drugs Drug Resist. 2012;2:58–75. doi: 10.1016/j.ijpddr.2011.10.001 24533264 10.1016/j.ijpddr.2011.10.001 PMC3862436

[60] Dicker AJ, Nisbet AJ, Skuce PJ. Gene expression changes in a P-glycoprotein (Tci-pgp-9) putatively associated with ivermectin resistance in Teladorsagia circumcincta. Int J Parasitol. 2011;41(9):935–42. doi: 10.1016/j.ijpara.2011.03.015 21683705 10.1016/j.ijpara.2011.03.015

[61] Arnold B, Corbett-Detig RB, Hartl D, Bomblies K. RADseq underestimates diversity and introduces genealogical biases due to nonrandom haplotype sampling. Mol Ecol. 2013;22(11):3179–90. doi: 10.1111/mec.12276 23551379 10.1111/mec.12276

[62] Ardelli BF. Transport proteins of the ABC systems superfamily and their role in drug action and resistance in nematodes. Parasitology international. 2013;62(6):639–46. doi: 10.1016/j.parint.2013.02.008 23474412 10.1016/j.parint.2013.02.008

[63] Tyden E, Skarin M, Hoglund J. Gene expression of ABC transporters in Cooperia oncophora after field and laboratory selection with macrocyclic lactones. Mol Biochem Parasitol. 2014;198(2):66–70. doi: 10.1016/j.molbiopara.2015.01.002 25619799 10.1016/j.molbiopara.2015.01.002

[64] De Graef J, Demeler J, Skuce P, Mitreva M, Von Samson-Himmelstjerna G, Vercruysse J, et al Gene expression analysis of ABC transporters in a resistant Cooperia oncophora isolate following in vivo and in vitro exposure to macrocyclic lactones. Parasitology. 2013;140(4):499–508. doi: 10.1017/S0031182012001849 23279803 10.1017/S0031182012001849 PMC3690601

[65] Laing ST, Ivens A, Butler V, Ravikumar SP, Laing R, Woods DJ, et al The transcriptional response of Caenorhabditis elegans to Ivermectin exposure identifies novel genes involved in the response to reduced food intake. PLoS One. 2012;7(2):e31367 doi: 10.1371/journal.pone.0031367 22348077 10.1371/journal.pone.0031367 PMC3279368

[66] Doyle SR, Bourguinat C, Nana-Djeunga HC, Kengne-Ouafo JA, Pion SDS, Bopda J, et al. Genome-wide analysis of ivermectin response by Onchocerca *volvulus* reveals that genetic drift and soft selective sweeps contribute to loss of drug sensitivity; 2016. Preprint. Availabel from bioRxiv. doi: 10.1101/094540 10.1371/journal.pntd.0005816 PMC5546710 28746337

[67] Wood WB. The nematode Caenorhabditis elegans. New York: Cold Spring Harbour Laboratory Press; 1988.

[68] Sulston J, Hodgkin J. Methods In: Wood WB, editor. The Nematode Caenorhabditis elegans. New York: Cold Spring Harbour Laboratory Press; 1988 p. 587–606.

[69] Tang YT, Gao X, Rosa BA, Abubucker S, Hallsworth-Pepin K, Martin J, et al Genome of the human hookworm Necator americanus. Nat Genet. 2014;46(3):261–9. doi: 10.1038/ng.2875 24441737 10.1038/ng.2875 PMC3978129

[70] Margulies M, Egholm M, Altman WE, Attiya S, Bader JS, Bemben LA, et al Genome sequencing in microfabricated high-density picolitre reactors. Nature. 2005;437(7057):376–80. doi: 10.1038/nature03959 16056220 10.1038/nature03959 PMC1464427

[71] Kent WJ. BLAT—the BLAST-like alignment tool. Genome Res. 2002;12(4):656–64. doi: 10.1101/gr.229202 11932250 10.1101/gr.229202 PMC187518

[72] Benson G. Tandem repeats finder: a program to analyze DNA sequences. Nucleic Acids Res. 1999;27(2):573–80. 9862982 10.1093/nar/27.2.573 PMC148217

[73] Lagesen K, Hallin P, Rodland EA, Staerfeldt HH, Rognes T, Ussery DW. RNAmmer: consistent and rapid annotation of ribosomal RNA genes. Nucleic Acids Res. 2007;35(9):3100–8. doi: 10.1093/nar/gkm160 17452365 10.1093/nar/gkm160 PMC1888812

[74] Lowe TM, Eddy SR. tRNAscan-SE: a program for improved detection of transfer RNA genes in genomic sequence. Nucleic Acids Res. 1997;25(5):955–64. 9023104 10.1093/nar/25.5.955 PMC146525

[75] Griffiths-Jones S, Bateman A, Marshall M, Khanna A, Eddy SR. Rfam: an RNA family database. Nucleic Acids Res. 2003;31(1):439–41. 12520045 10.1093/nar/gkg006 PMC165453

[76] Korf I. Gene finding in novel genomes. BMC Bioinformatics. 2004;5:59 doi: 10.1186/1471-2105-5-59 15144565 10.1186/1471-2105-5-59 PMC421630

[77] Salamov AA, Solovyev VV. Ab initio gene finding in Drosophila genomic DNA. Genome Res. 2000;10(4):516–22. 10779491 10.1101/gr.10.4.516 PMC310882

[78] Stanke M, Diekhans M, Baertsch R, Haussler D. Using native and syntenically mapped cDNA alignments to improve de novo gene finding. Bioinformatics. 2008;24(5):637–44. doi: 10.1093/bioinformatics/btn013 18218656 10.1093/bioinformatics/btn013

[79] Cantarel BL, Korf I, Robb SM, Parra G, Ross E, Moore B, et al MAKER: an easy-to-use annotation pipeline designed for emerging model organism genomes. Genome Res. 2008;18(1):188–96. doi: 10.1101/gr.6743907 18025269 10.1101/gr.6743907 PMC2134774

[80] Boeckmann B, Bairoch A, Apweiler R, Blatter MC, Estreicher A, Gasteiger E, et al The SWISS-PROT protein knowledgebase and its supplement TrEMBL in 2003. Nucleic Acids Res. 2003;31(1):365–70. 12520024 10.1093/nar/gkg095 PMC165542

[81] Finn RD, Mistry J, Schuster-Böckler B, Griffiths-Jones S, Hollich V, Lassmann T, et al Pfam: clans, web tools and services. Nucleic Acids Res. 2006;34(Database issue):D247–51. doi: 10.1093/nar/gkj149 16381856 10.1093/nar/gkj149 PMC1347511

[82] Marchler-Bauer A, Lu S, Anderson JB, Chitsaz F, Derbyshire MK, DeWeese-Scott C, et al CDD: a Conserved Domain Database for the functional annotation of proteins. Nucleic Acids Res. 2011;39(Database issue):D225–9. doi: 10.1093/nar/gkq1189 21109532 10.1093/nar/gkq1189 PMC3013737

[83] Kanehisa M, Goto S, Sato Y, Furumichi M, Tanabe M. KEGG for integration and interpretation of large-scale molecular data sets. Nucleic Acids Res. 2012;40(Database issue):D109–14. doi: 10.1093/nar/gkr988 22080510 10.1093/nar/gkr988 PMC3245020

[84] Zdobnov EM, Apweiler R. InterProScan—an integration platform for the signature-recognition methods in InterPro. Bioinformatics. 2001;17(9):847–8. 11590104 10.1093/bioinformatics/17.9.847

[85] Kall L, Krogh A, Sonnhammer EL. A combined transmembrane topology and signal peptide prediction method. Journal of molecular biology. 2004;338(5):1027–36. doi: 10.1016/j.jmb.2004.03.016 15111065 10.1016/j.jmb.2004.03.016

[86] Bendtsen JD, Jensen LJ, Blom N, Von Heijne G, Brunak S. Feature-based prediction of non-classical and leaderless protein secretion. Protein Eng Des Sel. 2004;17(4):349–56. doi: 10.1093/protein/gzh037 15115854 10.1093/protein/gzh037

[87] Tamura K, Stecher G, Peterson D, Filipski A, Kumar S. MEGA6: Molecular Evolutionary Genetics Analysis version 6.0. Mol Biol Evol. 2013;30(12):2725–9. doi: 10.1093/molbev/mst197 24132122 10.1093/molbev/mst197 PMC3840312

[88] Wang Z, Abubucker S, Martin J, Wilson RK, Hawdon J, Mitreva M. Characterizing Ancylostoma caninum transcriptome and exploring nematode parasitic adaptation. BMC Genomics. 2010;11:307 doi: 10.1186/1471-2164-11-307 20470405 10.1186/1471-2164-11-307 PMC2882930

[89] Dobin A, Davis CA, Schlesinger F, Drenkow J, Zaleski C, Jha S, et al STAR: ultrafast universal RNA-seq aligner. Bioinformatics. 2013;29(1):15–21. doi: 10.1093/bioinformatics/bts635 23104886 10.1093/bioinformatics/bts635 PMC3530905

[90] Engstrom PG, Steijger T, Sipos B, Grant GR, Kahles A, Consortium R, et al Systematic evaluation of spliced alignment programs for RNA-seq data. Nature methods. 2013;10(12):1185–91. doi: 10.1038/nmeth.2722 24185836 10.1038/nmeth.2722 PMC4018468

[91] Feng J, Meyer CA, Wang Q, Liu JS, Shirley Liu X, Zhang Y. GFOLD: a generalized fold change for ranking differentially expressed genes from RNA-seq data. Bioinformatics. 2012;28(21):2782–8. doi: 10.1093/bioinformatics/bts515 22923299 10.1093/bioinformatics/bts515

[92] Bolger AM, Lohse M, Usadel B. Trimmomatic: a flexible trimmer for Illumina sequence data. Bioinformatics. 2014;30(15):2114–20. doi: 10.1093/bioinformatics/btu170 24695404 10.1093/bioinformatics/btu170 PMC4103590

[93] Li H. Aligning sequence reads, clone sequences and assembly contigs with BWA-MEM. arXiv:13033997v2 [q-bioGN]. 2013.

[94] McKenna A, Hanna M, Banks E, Sivachenko A, Cibulskis K, Kernytsky A, et al The Genome Analysis Toolkit: a MapReduce framework for analyzing next-generation DNA sequencing data. Genome Res. 2010;20(9):1297–303. doi: 10.1101/gr.107524.110 20644199 10.1101/gr.107524.110 PMC2928508

[95] Kofler R, Pandey RV, Schlotterer C. PoPoolation2: identifying differentiation between populations using sequencing of pooled DNA samples (Pool-Seq). Bioinformatics. 2011;27(24):3435–6. doi: 10.1093/bioinformatics/btr589 22025480 10.1093/bioinformatics/btr589 PMC3232374

[96] Gasser T, Kneip A, Kohler W. A Flexible and Fast Method for Automatic Smoothing. J Am Stat Assoc. 1991;86(415):643–52.

[97] Herrmann E. Local bandwidth choice in kernel regression estimation. J Comput Graph Stat. 1997;6(1):35–54.

[98] Beissbarth T, Speed TP. GOstat: find statistically overrepresented Gene Ontologies within a group of genes. Bioinformatics. 2004;20(9):1464–5. doi: 10.1093/bioinformatics/bth088 14962934 10.1093/bioinformatics/bth088

[99] Nijveen H, van Kaauwen M, Esselink DG, Hoegen B, Vosman B. QualitySNPng: a user-friendly SNP detection and visualization tool. Nucleic Acids Res. 2013;41(Web Server issue):W587–90. doi: 10.1093/nar/gkt333 23632165 10.1093/nar/gkt333 PMC3692117

[100] Tang J, Vosman B, Voorrips RE, van der Linden CG, Leunissen JA. QualitySNP: a pipeline for detecting single nucleotide polymorphisms and insertions/deletions in EST data from diploid and polyploid species. BMC Bioinformatics. 2006;7:438 doi: 10.1186/1471-2105-7-438 17029635 10.1186/1471-2105-7-438 PMC1618865

[101] Li H. FermiKit: assembly-based variant calling for Illumina resequencing data. Bioinformatics. 2015;31(22):3694–6. doi: 10.1093/bioinformatics/btv440 26220959 10.1093/bioinformatics/btv440 PMC4757955

[102] Xie C, Tammi MT. CNV-seq, a new method to detect copy number variation using high-throughput sequencing. BMC Bioinformatics. 2009;10:80 doi: 10.1186/1471-2105-10-80 19267900 10.1186/1471-2105-10-80 PMC2667514

[103] Kofler R, Orozco-terWengel P, De Maio N, Pandey RV, Nolte V, Futschik A, et al PoPoolation: a toolbox for population genetic analysis of next generation sequencing data from pooled individuals. PLoS One. 2011;6(1):e15925 doi: 10.1371/journal.pone.0015925 21253599 10.1371/journal.pone.0015925 PMC3017084

[104] Lennon NJ, Lintner RE, Anderson S, Alvarez P, Barry A, Brockman W, et al A scalable, fully automated process for construction of sequence-ready barcoded libraries for 454. Genome Biol. 2010;11(2):R15 doi: 10.1186/gb-2010-11-2-r15 20137071 10.1186/gb-2010-11-2-r15 PMC2872875

[105] Catchen J, Hohenlohe PA, Bassham S, Amores A, Cresko WA. Stacks: an analysis tool set for population genomics. Mol Ecol. 2013;22(11):3124–40. doi: 10.1111/mec.12354 23701397 10.1111/mec.12354 PMC3936987

[106] Weir BS, Cockerham CC. Estimating F-Statistics for the Analysis of Population Structure. Evolution. 1984;38(6):1358–70. doi: 10.1111/j.1558-5646.1984.tb05657.x 28563791 10.1111/j.1558-5646.1984.tb05657.x

[107] Danecek P, Auton A, Abecasis G, Albers CA, Banks E, DePristo MA, et al The variant call format and VCFtools. Bioinformatics. 2011;27(15):2156–8. doi: 10.1093/bioinformatics/btr330 21653522 10.1093/bioinformatics/btr330 PMC3137218

[108] Humbert J-F, Elard L. A simple PCR method for rapidly detecting defined point mutations. Technical Tips Online. 1997;2(1):48–9.

