# Supplementary material for: Genomic introgression mapping of field-derived multiple-anthelmintic resistance in Teladorsagia circumcincta
Source: PLoS Genet. 2017 Jun 23;13(6):e1006857. doi: 10.1371/journal.pgen.1006857 (PMC5507320; doi:10.1371/journal.pgen.1006857)
Supplement: S1 Fig — (PDF) [file pgen.1006857.s001.pdf]

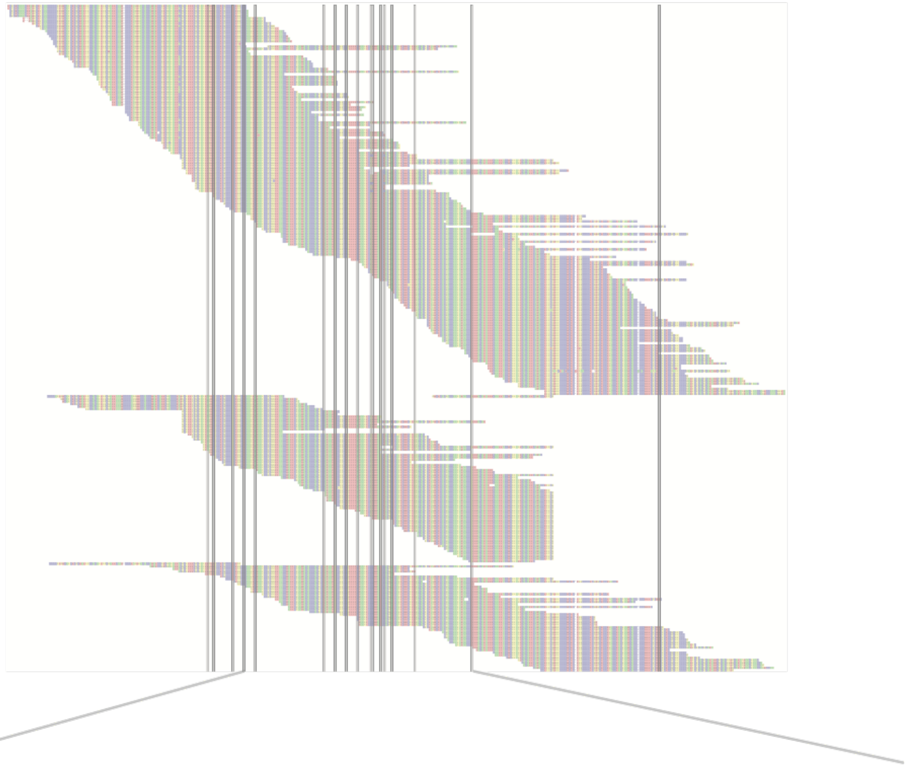

Coordinate	443055	443061	443097	443103	443109	443115	443122	443123	443127	443129	443133	443145	443175
Reference allele	T	T	A	G	T	T	G	A	A	T	C	A	A
Haplotype 2	T	T	A	G	T	T	G	A	A	A	C	A	A
Haplotype 1	A	C	T	A	A	C	G	A	G	A	T	G	G
Haplotype 3	T	T	A	G	T	T	T	T	A	T	C	A	A

S1 Fig. Reconstructed haplotypes of β-tubulin *isotype-1* in *Teladorsagia circumcincta* RS^3 population. Read-backed phasing approach was applied over the exonic region harboring E198L (GAa/TTa) and F200Y (tTc/tAc) variants located at Cont53:443122-443123 and Cont53:443129, respectively.
