# Supplementary material for: Genomic introgression mapping of field-derived multiple-anthelmintic resistance in Teladorsagia circumcincta
Source: PLoS Genet. 2017 Jun 23;13(6):e1006857. doi: 10.1371/journal.pgen.1006857 (PMC5507320; doi:10.1371/journal.pgen.1006857)
Supplement: S2 Fig — (PDF) [file pgen.1006857.s002.pdf]

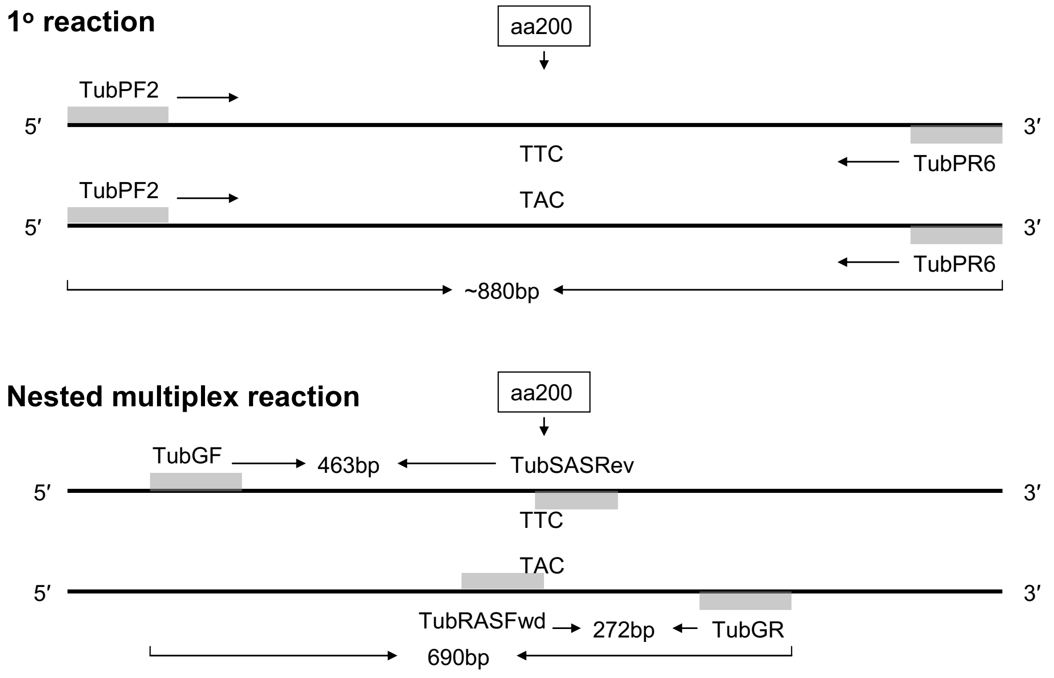

S2 Fig. Allele-specific multiplex PCR strategy to assess the presence of a F200Y (tTc/tAc) substitution in the β-tubulin *isotype-1* gene in individual male *Teladorsagia circumcincta*.
