# Supplementary material for: Genomic introgression mapping of field-derived multiple-anthelmintic resistance in Teladorsagia circumcincta
Source: PLoS Genet. 2017 Jun 23;13(6):e1006857. doi: 10.1371/journal.pgen.1006857 (PMC5507320; doi:10.1371/journal.pgen.1006857)
Supplement: S3 Fig — (PDF) [file pgen.1006857.s003.pdf]

```
                  1                                                         60
Sinbred [S1a]     TTCGCGAGGAGTATCCGGATAGAATCATGGCTTCATTCTCCGTTGTTCCATCACCAAAGG
Sinbred [S1b]     -----------------------------------TTC----------------------
RS3 [R2]          -----------------------------------TTC----------------------
RS3 [R1c]         -----------------------------------TTC----------------------
RS3 [R1b]         -----------------------------------TTC--------------G-------
RS3 [R1a]         -----------------------------------TTC--------------G-------

                  61                                                       120
Sinbred [S1a]     TAATGTATCCC··ACAGCAGTCCTTTTT·GAGATCGTATTTACAGGTTCTCGAGGTGCAA
Sinbred [S1b]     -----------TA----T----------·---------G---------------------
RS3 [R2]          -----------TA----T----------·---------G---------------------
RS3 [R1c]         -----------TA----T----------·---------G---------------------
RS3 [R1b]         ----T----T-TG----TC---------C---------G---------------------
RS3 [R1a]         ----T----T-TG----TC---------C---------G---------------------

                  121                                                      180
Sinbred [S1a]     TCCATTTTACGAACTCTTCACATTGATGCGCAACCGTGAAATGTGCGAAGAAGTTATGTT
Sinbred [S1b]     -------·----G-----------------------------------------------
RS3 [R2]          -------·----G---------------------T-------------------------
RS3 [R1c]         -------·----G---------------------T-------------------------
RS3 [R1b]         C-------CG---T----T----CA----TTT--A-----G-------C--T--------
RS3 [R1a]         C-------CG---T----T----CA----TTT--A-----G-------C--T--------

                  181                                                      240
Sinbred [S1a]     TATAGGTTTCCGATACCGTTGTGGAACCTTACAATGCCACTCTTTCTGTACACCAGTTGG
Sinbred [S1b]     ------------------------------------------------------------
RS3 [R2]          ------------------------------------------------------------
RS3 [R1c]         ------------------------------------------------------------
RS3 [R1b]         -G-----A----------------------------------------------------
RS3 [R1a]         -G-----A-----C-----------------------------------T-----A----

                  241                                                      300
Sinbred [S1a]     TTGAAAATACCGATGAAACATTCTGCATCGATAATGAAGCTCTGTACGATATCTGCTTCC
Sinbred [S1b]     --------------GAA---TTC-------------------------------------
RS3 [R2]          --------------TTA---TTC-------------------------------------
RS3 [R1c]         --------------GAA---TAC-------------------------------------
RS3 [R1b]         --------------GAA---TAC-------------------------------------
RS3 [R1a]         -A-----C------GAA--GTAC-T------------G----------------------

                  301                                                      360
Sinbred [S1a]     GCACCTTAAAACTCACAAATCCAACTTATGGCGATCTCAATCACTTAGGTGAGGTTTAAT
Sinbred [S1b]     ---------------------------------------------------------T--
RS3 [R2]          ---------------------------------------------------------T--
RS3 [R1c]         ---------------------------------------------------------T--
RS3 [R1b]         ---------------------------------------------------------T--
RS3 [R1a]         -------G-------------------------------------------C-TAC--T-

                  361                                                      420
Sinbred [S1a]     ATAGGTTCTATGAATATTAGCTCACTGCCATCTTTT·TAAATGTATTGTATTGTGGTTTT
Sinbred [S1b]     ------------------------------------·-----------------------
RS3 [R2]          -------T-----G--------T-------------·--------A--------------
RS3 [R1c]         -------T-----G--------T-------------·-----------------------
RS3 [R1b]         -------T-----G--------T-----------C-·-----------------------
RS3 [R1a]         ---A-CCT-·-A-G--------T-----------C-C----C-C---------C------
```

S3 Fig. ClustalW multiple alignment of selected gDNA sequence variants from the central region of the β-tubulin *isotype-1* gene from S_{inbred} and RS^3 worms showing amino acid codon positions 167, 198 and 200 (bold), SNPs and positions of introns (shaded areas).

For the purposes of genotyping individual male worms from S_{inbred} and RS^3 populations, three "alleles" were recognized (S_1, R_1 and R_2) and allele-specific PCR strategies designed to distinguish between these. In both S_1 and R_2 alleles, codon 200 encoded Phe, but in the case of the R_2 allele a mutation at codon 198 resulted in a Glu/Leu substitution. In all R_1 variants amino acid codon 200 encoded Tyr. Note that in order to accommodate the presence of SNPs in sequence flanking codon 200 in variants of the BZ resistant R_1 allele, the allele-specific primer TubRASFwd was designed to be degenerate in three nucleotide positions (see Methods).
