# Supplementary material for: Genomic introgression mapping of field-derived multiple-anthelmintic resistance in Teladorsagia circumcincta
Source: PLoS Genet. 2017 Jun 23;13(6):e1006857. doi: 10.1371/journal.pgen.1006857 (PMC5507320; doi:10.1371/journal.pgen.1006857)
Supplement: S4 Fig — (PDF) [file pgen.1006857.s004.pdf]

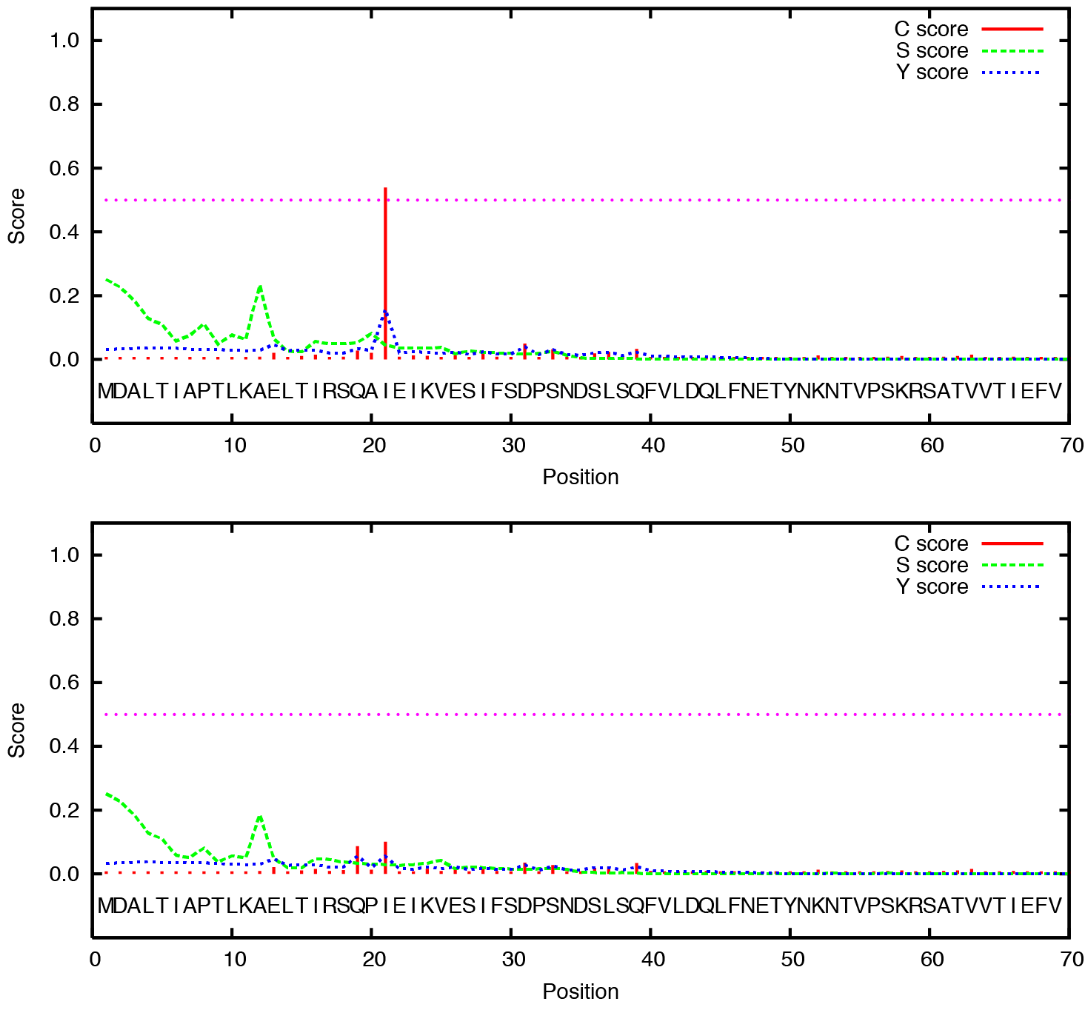

S4 Fig. Prediction of signal peptide cleavage site in Tci-LGC-54 (TELCIR_00170).

Prediction was done using SignalP 3.0 (neural network). Substitution of Ala (upper panel) for Pro (lower panel) at position 20 results in the loss of a predicted cleavage site between amino acid 20-21. C-score is the “cleavage site” score.
