# Supplementary material for: Genomic introgression mapping of field-derived multiple-anthelmintic resistance in Teladorsagia circumcincta
Source: PLoS Genet. 2017 Jun 23;13(6):e1006857. doi: 10.1371/journal.pgen.1006857 (PMC5507320; doi:10.1371/journal.pgen.1006857)
Supplement: S5 Fig — (PDF) [file pgen.1006857.s005.pdf]

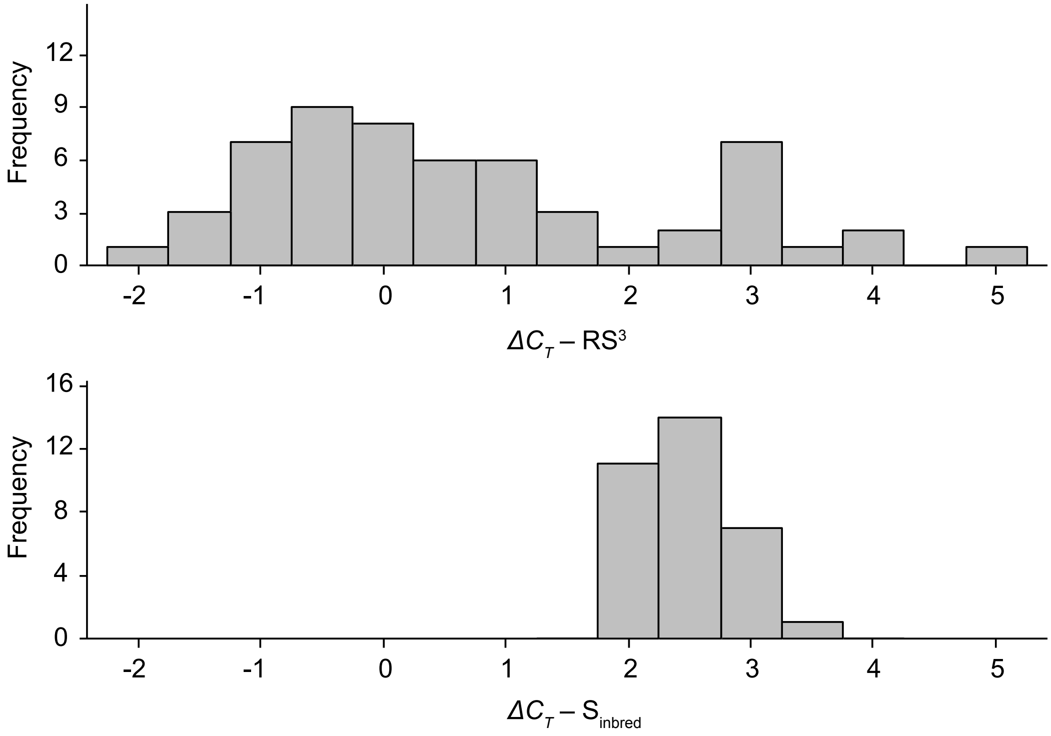

S5 Fig. Distribution of ΔC_T values of individual male worms from the *T. circumcincta* strains RS^3 and S_{inbred}. Note that ΔC_T values represent the difference in the number of PCR cycles required to reach a fixed DNA threshold for the target (*Tci-pgp-9*) and reference genes (*Tci-tbb-iso-1*). Thus low ΔC_T values indicate high template concentrations of target relative to the reference gene while high ΔC_T values indicate low template concentrations of target relative to the reference gene.
