# Supplementary material for: Genomic introgression mapping of field-derived multiple-anthelmintic resistance in Teladorsagia circumcincta
Source: PLoS Genet. 2017 Jun 23;13(6):e1006857. doi: 10.1371/journal.pgen.1006857 (PMC5507320; doi:10.1371/journal.pgen.1006857)
Supplement: S6 Fig — (PDF) [file pgen.1006857.s006.pdf]

```
        1                                                                                                                    120
Hap 2   ATGCTGTTTAACACAACGATTGAACAGGTTTGTACTCACGAAAGTTTCGATCAAAAGAAAAATGGAAAAATTAAAAATAACCCATTCCTTTCAGAATATCCGCTATGGACGTGAAAAAGT
Hap 1   ATGCTGTTTAATACAACGATCGAACAGGTTTGTTTT······TGGAGAGATCTGATGAATAGAAAAAAAAAAGGAAAATGGTTAATCTCATTCAGAATATCCGTTATGGACGTGAAAAAGT
Hap 4   ATGCTGTTCAACACGACCATTGAACAGGTTTGTTTTC·GGGGAGGTCTTATCAACAGAAAA·····AAAAAGGAAAATGGCTAATCTCATTCAGAATATCCGATATGGACGTGAAAAAGT
Hap 3   ATGCTGTTCAACACGACCATTGAACAGGTTTGTTTTC·GGGGAGGTCTTATCAACAGAAAA·····AAAAAGGAAAATGGCTAATCTCATTCAGAATATCCGATATGGACGTGAAAAAGT
Hap 9   ATGCTTTTCAACACAACGATTGAACAGGTTTGTTTTT·GAAGAGGTCTGATCAATAGAAAAAAGGAAAAATTGAAAATGACGTATATCTTTCAGAATATTCGCTATGGACGTGAAAAAGT
Hap 7   ATGCTGTTTAACACAACGATTGAACAGGTTTGTTCTCACGAGAGCTTTAATCCGAAGAAAAATGGGAAGATGGAAAATAACTCGGTCCTTTCAGGATATCCGCTATGGACGTGAAAAAGT
Hap 8   ATGCTGTTCAACACAACGATTGAACAGGTTTGTTCTCACGAGAGGTTTGATCAAAAGAAAAATGGAAAAACTGAAAATAACCCATTCCTTTCAGAACATCCGCTATGGACGTGAAAAAGT
Hap 10  ATGCTCTTTAACACGACGATTGAACAGGTTCGTTCCCACGAGAGCTTTGATCAAACGAAAAATGG·AAAACTGAAAATAACCCATTCCTTTCAGAACATCCGCTATGGACGTGAAAAAGT
Hap 6   ATGCTGTTTAACACAACGATTGAACAGGTTTGTTCTCACGAGAGCTTTGATCCAAAGAAAAATGGAAAGATGGAAAATAACTCGTTCCTTTTAGAATATCCGCTATGGACGTGAAAAAGT
Hap 5   ATGCTGTTTAACACAACGATTGAACAGGTTTGTTCTCACGAGAGCTTTGATCCAAAGAAAAATGGAAAGATGGAAAATAACTCGTTCCTTTTAGAATATCCGCTATGGACGTGAAAAAGT
         M  L  F  N  T  T  I  E  Q                                                                      N  I  R  Y  G  R  E  K  V

        121                                                                                                                  240
Hap 2   CACAGATGCTGAAATCACGGCGGCACTCCGTAAAGCAAACGCCTACAATTTTGTGCAGTCATTCCCTGACGTGAGTTGGAACCTCGTGCTGTCGGTGTTAAAAGCAGTGATGGTAATGGC
Hap 1   CACAGATGCTGAAATCACAGCGGCACTCCGCAAAGCAAACGCCTACAATTTTGTGCAGTCGTTCCCCGACGTGAGTTGGAACCTCGTATTGTCGATCTTAAAGGTGGTGATCGCAATGGG
Hap 4   CACAGATGCTGAAATCACGGCGGCACTCCGCAAAGCAAACGCCTACAATTTTGTGCAGTCATTCCCTGACGTGAGTTGGAATCTCGTGCTGTTGA···TAGATGCAGTGAGTGCAATAGC
Hap 3   CACAGATGCTGAAATCACGGCGGCACTCCGCAAAGCAAACGCCTACAATTTTGTGCAGTCATTCCCTGACGTGAGTTGGAATCTCGTGCTGTTGA···TAGATGCAGTGAGTGCAATAGC
Hap 9   CACAGACGCTGAAATAACGGCCGCACTCCGTAAAGCAAACGCCTACAATTTTGTGCAGTCGTTCCCTGACGTGAGTTGGAACCTCGTGCTGTTGACGTTAAAGGCAGTAAGTGCAATGGC
Hap 7   CACAGATGCTGAAATCACAGCGGCACTCCGCAAAGCGAACGCCTATAACTTTGTACAGTCGTTCCCTGACGTGAGTTGGAACCTCGTGCTGTTGACGTTAAAGGCAGTAAGTGCAATGGC
Hap 8   CACAGATGCTGAAATCACGGCGGCACTCCGTAAAGCGAACGCCTACAATTTTGTGCAGTCATTCCCTGACGTGAGTTGGAACCTCGTGCTGTCGACCTTAAAGGCAGTGAGGGCAATGCC
Hap 10  CACAGATGCTGAAATCACGGCAGCACTCCGTAAAGCGAACGCCTACAATTTTGTGCAGTCATTCCCTGACGTGAGTTGGAACCTCGTGTTGTCGGTGTTAAAGGCAGTCAGTGCAATGGC
Hap 6   CACAGATGCTGAAATCACAGCGGCACTCCGCAAAGCGAACGCCTATAACTTTGTACAGTCGTTCCCTGACGTGAGTTGGAACCTTGTGCTATTGATGTTAAAGGCAGTGATTGCAATGGC
Hap 5   CACAGATGCTGAAATCACAGCGGCACTCCGCAAAGCGAACGCCTATAACTTTGTACAGTCGTTCCCTGACGTGAGTTGGAACCTCTTGCTGTTGATGTTAGAGGCAGTGAGTGCAATACC
          T  D  A  E  I  T  A  A  L  R  K  A  N  A  Y  N  F  V  Q  S  F  P  D

        241                                                                                                                  360
Hap 2   TG··········AGAGGCGGGTCGTCGCTGGAAAGGGTTGCAAGAGGGGGTGGTGGGAGGG··GGTGTGGTCACACCGCCCCTCCCCCCTCCTCCCACA·····················  [IBD77RAS11]
Hap 1   TG··········ACGTCCGGGGTTTCGAGGACGTTGGTGGAACAACGCTGCAAGAAAAAGGGCAGGAGGGGTATGGTCAGACACCCCCTCCCCTCCCACA·····················
Hap 4   TGGT····AGGCGGAGTCAGTCTAAGAAAG····TGGTGGAATGGCGCCGGGAAGGAAGGAACCGGCAGGGTTTTCTTTCCAATCCCCCCAACCCCTTTACACGGGTAACCCAAAAAAAA
Hap 3   TGGT····AGGCGGAGTCAGTCTAAGAAAG····TGGTGGAATGGCGCTGGGAAGG·AGAGAGCGGCAGGGTGTCGTT··CAATCCCC··AACGCCCTTACACGGGTACCCGACAAGA··  [IBD77RAS12]
Hap 9   TGGT····AAGCGGAGCTAGTTTGAGGAAGCCGTTGGTGGAACGGAACTTGGAAGGAGAGAGCGGGAGGATGTCGTAGGATCTTCCCCGA··CGTTATCACACGGGTACCCGACAACAGG  [IBD77RAS9A]
Hap 7   TGGT····AAGCGGAGTTAGTTCGAAGAAACCGTTGGTGGAACGGAACTTGGAAGGAGAAAACCGGAAGGTGTCCTTAGATTCTCTCCCAATGTTTATCACACCGGTACCCGACAATAGG  [IBD77RAS2]
Hap 8   TGGT····AGGCGGATTCAGTTTGAGAAAGTCAATGGTGGAACGGTGTTGAGAGGGAGAGGGCAGGAGGGTGTCATAGGATCAGTCCCTAA·CGCCCTTACACGGGTACCCGACAAGAGG  [IBD77RAS8A]
Hap 10  TGGT····GGGGGAAGTTAGTTTGAGGAAGTCATTGGTGGAACGGCTCTGGGAAGGAGAGCGCGGAGCGGTGTTGTAGGACTACTCCCCAAT·GCCCTCGCACGGGTACCCGACAAGAGG  [IBD77RAS10C]
Hap 6   TGGTGGGGGGGGGGAGTTAGTTTGAGGAAGTCATTGGTGGAACGGCGCTGGGAAGGAGTGGGCGGGAGGGTGTCGTAGGACCACTTCCCAA·CGCCCTAACACCGGTACCCGACAAGAGG
Hap 5   TGGT····GGGCGGAGTCA································GAAGGAGAGGGCGGGACGGCGTCGTAGGACTACTGCCCAA·CGCTTTTACACGGGTACCCGACAAGAGG

        361                                                                                                                  480
Hap 2   ························································································································
Hap 1   ························································································································
Hap 4   AGCCCCTCCCCGGGGGGGGAGAAGGCAGATTTCAGACAGTTCAGCGGACGGAGTCGG··AGCGGGAACCTGGGAAGTACTATAGTGTAGCCTATTTAAATTGACGTTGA·········  [IBD77RAS3]
Hap 3   ····GGCCCGTCGCGCGGCGAGAAGGCAGATCTCAGACAGGTCAGCGGAC·GAGTCGG··AGCGCG·ATCTGGGACGTACTATAGTGTAGCCTATTTAAATTGACGTTGA·········
Hap 9   CCCGTCGCGCGGCGAGAAGGCATATCTACAGACGTGTCAGCGGACGAGTAGGAGCGCGTATTGTGCGATGTGGGACGTACTAT·GTATAGTATATTTGAGCTACCGCTGAATCGCTACAG
Hap 7   CCCGTCGCGCGGCGAAAAGGTTGATCTACAGAAGTTTCAGCGGAGGAGTAGGAGCGCGTATTGTGNGATGTGGGACGTACTAT·GTATAGTATATTTGAGCTACCGNTGATTCGTTACAG
Hap 8   CCCGTCGCGCGGCGAGAAGGCAGATCTACATACAGGTCAGCGGACGAGTCCGAGCGCGTATTGTGCGATGTGGGACGTAC····TATAGTATATTTAAATTACCGCTGAATCGCTACAG
Hap 10  CCCGTCGCGTAGCGAGAAGGCAGATCTACGGACAGGTCAGCGGACGAG···CAGCGCGTATTGTGCGATGTGGGACGTACTATAGTATAGTATATTTAAATTGACGCTGAATCGCTACAG
Hap 6   CCCGTCGCGCAGCGAGAAGGCGGATCTACAGACGGGTCAGCGGATGATTCGCAGCGCGCATTGTGTGATGTGGGA··TACTATAGTAAAGTCTATTTAAATTGACGCTGAATCGCCACAG
Hap 5   CCCGTCGCGCGGCGAGAAGGCAAATCTACGGACAGGTCAGCGGACGAGCA···CGCGTATTTTGCGATGTCGGACGTACTATAGTATAGTCTATTAAATT····CGAGATCGCCACAG←
```

```
         481                                                                                                                    600
Hap 2    ························································································ACGCCGTTCCACCAGTGACGTTATTGAATTGGTTTCGCCTCTGAACTG
Hap 1    ························································································ACGCCCTTCCACCAATGACGTTCCTGAATCAGTTCCGCCTCTCAGCTG
Hap 4    ·················CCCTTCACTTTTCTGCGTCCCTGCCTTCGCACCGCGGGCAGGCCTCCTGTCGGGTACCTTCTCACACCAATGACGTTCCTGAGTTGACTCCGCCTCTCTGCTG
Hap 3    ·················CCCTTCACCTCTCTGCGTCCCTGCCTTCGCACCGCGGGCAGGCCTCCTGTCGGGTACCTTCTCACACCAATGACGTTCCTGAGTTGACTCCGCCTCTCTGCTG
Hap 9    TACGCGCACAGGCACGTCCCCTCACTTGTCTGCGTCCTT···········GCGGGCAGGCCTCCTGTCGGGTACCTCCT·ACACCAATGAGATCACTTAACTCCACCTCCTAGCTGTCGC
Hap 7    TACGCGCACAGGCAAGTCCCTTCACTTGTCTGCGTCCTT···········GCGGGCAGGCTTCCTGTCGGGTACTTCCT·ACACCAATGAGATCACTTAACTCCACCTCCTAGCTGTCGC
Hap 8    TACGCGCACAGGCACGTCCCCTCACTTGTCTGCGTCCCT···········GTGGGCAGGCCTCCTGTCGGGTACTTCCT·ACACCAATGAGATCCCTTAACTCCACCTCCCAGCTGTCGC
Hap 10   TACGCGCACAGGCACGTCCCCTCACCTGTCTGCGTCCCTGCCATTGCATCGCGGGCAGGCCTCCTGTCGGGTACTTCCT·ACACCAATGAGGTCTCTTAACTCCGCCTCCCAGCTGTCGC
Hap 6    TACGCGCTCAGGCACGTCCCCTCGCCTGTCTGCGTCCCTGCCTTCGCACTGCGGGCAGGCC·CCGTCCGGTACCCCCTT·ACACCAGTGAAGTCCTCTGACTCTGCCTTTCAGCTGTTGC [IBD77RAS5B]
Hap 5   ←TACGCGCTCAGGCACATCCGCTCACCTGTCTGCGTCCCTGCCTTCGCACCGTGGGCAGGCCTCCTGTCGGGTACCTTCTCACACCAATGACGTTCCTGAATTGACTCCGCCTCTCTGCTG [IBD77RAS4]

         601                                                  654
Hap 2    CTGCAATCGCTACTGCTGGTTTCTCGTAACGAAGTCAAGC··TCGTTTCAGGGAATCTAC
Hap 1    CTGCAATAACTACTGCTGATTCCTCGT·TGAAAGGTGAGC··TTATTTCAGGGAATCTAC [IBD77RAS7]
Hap 4    CTGCAATCACTACTGCTTGCTTCCCGTAACAAAGTCAAGC··TCGTTTCAGGGAATTTAC
Hap 3    CTGCAATCACTACTGCTTGCTTCCCGTAACAAAGTCAAGC··TCGTTTCAGGGAATTTAC
Hap 9    AATCACTGCTCTAAACCACTGCCCCTCAACGAGCTGGACC··TTTTTTCAGGGT······
Hap 7    AATCACTGCCCTAAACCACTGCCCCTCAACGAGCTGGACC··TTTTTTCAGGGAATCTAC
Hap 8    AATCACTGCTCTAAACTACTGCCCCTCAACGAGCTGGACCTTTTTTTTCAGGGA······
Hap 10   AATCACTGCTCTAAACTACTGCCCCTCAACGAGCGAGAAC·TTTTCTCCAGGGA······
Hap 6    AACCACTGCTCTCAAACTACTGCCCTTAACGAGCTAGATC··TTCTTTCAGGGGATCTAC
Hap 5    CTGCAATCACTACTGCTTGCTTCCCGTAACAAAGTCAAGC··TCGTTTCAGGGAATTTAC
                                                          G  I  Y
```

S6 Fig. Multiple-alignment of partial sequences of *Tci-pgp-9-IBDA* haplotypes showing introns (shaded), amino acid translation (underlined) and locations of allele-specific reverse primers used in genotyping reactions (boxed). *Tci-pgp-9-IBDA* haplotypes were identified based on PCR clones (n=66) amplified from gDNA preparations. “Allele-specific” primers were designed to differentiate between each of the haplotypes using a nested PCR strategy to allow genotyping of individual male worms from the S_{inbred} and RS^3 strains of *T. circumcincta*.
