# Supplementary material for: Genomic introgression mapping of field-derived multiple-anthelmintic resistance in Teladorsagia circumcincta
Source: PLoS Genet. 2017 Jun 23;13(6):e1006857. doi: 10.1371/journal.pgen.1006857 (PMC5507320; doi:10.1371/journal.pgen.1006857)
Supplement: S7 Fig — (PDF) [file pgen.1006857.s007.pdf]

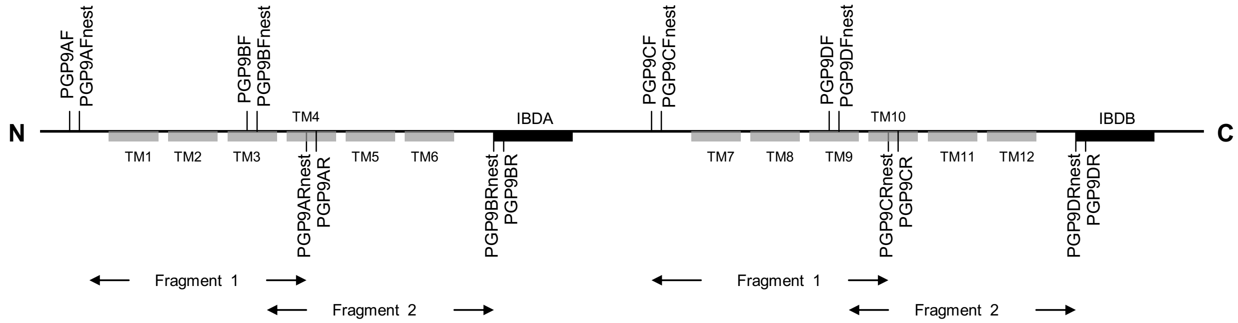


**S7 Fig. Approximate locations of the oligonucleotide primer sites used to amplify cDNA fragments encoding the N-terminal and C-terminal transmembrane regions of Tci-PGP-9 protein molecules from worms from the $S_{inbred}$ and $RS^3$ strains.** Forward primers are shown above the line depicting the Tci-PGP-9 molecule while reverse primers are shown below it. Grey shaded blocks indicate the relative positions of the transmembrane domains (TM1 – TM12), while black blocks indicate positions of the inter-nucleotide binding domains (IBDA & IBDB). The N-terminal fragments 1 and 2 incorporated aa34 – aa212 and aa189 – aa420 respectively while the C-terminal fragments 1 and 2 incorporated aa662 – aa874 and aa846 – aa1069 respectively.
