# Supplementary material for: Genomic introgression mapping of field-derived multiple-anthelmintic resistance in Teladorsagia circumcincta
Source: PLoS Genet. 2017 Jun 23;13(6):e1006857. doi: 10.1371/journal.pgen.1006857 (PMC5507320; doi:10.1371/journal.pgen.1006857)
Supplement: S8 Fig — (PDF) [file pgen.1006857.s008.pdf]

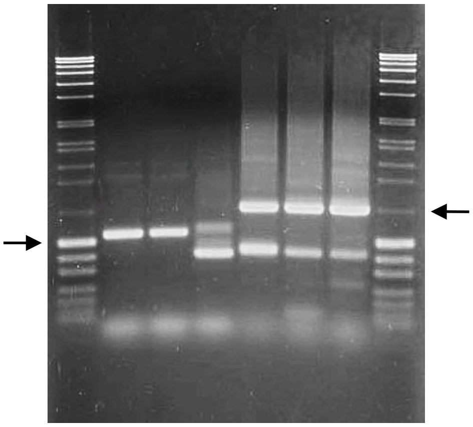

**S8 Fig. Agarose gel showing cDNA products amplified by PCR from the N-terminal transmembrane region of *Tci-pgp-9*.** Lanes 2 & 3 – PGP9AFnest/PGP9ARnest products, encoding Tci-PGP-9 transmembrane domains 1 - 3 (aa34 – aa212), amplified from separate pools of cDNA template derived from two different generations of $S_{inbred}$ worms; lane 4 – equivalent PGP9AFnest/PGP9ARnest products amplified from cDNA template derived from $RS^3$ worms; lanes 5 & 6 – PGP9BFnest/PGP9BRnest products, encoding Tci-PGP-9 transmembrane domains 4 - 6 (aa189 – aa420), amplified from cDNA template derived from $S_{inbred}$ worms as above; lane 7 – equivalent PGP9BFnest/ PGP9BRnest products amplified from cDNA template derived from $RS^3$ worms.
