# Supplementary material for: Genomic introgression mapping of field-derived multiple-anthelmintic resistance in Teladorsagia circumcincta
Source: PLoS Genet. 2017 Jun 23;13(6):e1006857. doi: 10.1371/journal.pgen.1006857 (PMC5507320; doi:10.1371/journal.pgen.1006857)
Supplement: S9 Fig — (PDF) [file pgen.1006857.s009.pdf]

```
   1  MGFLKKNGKV ADSKGQEDSQ IEEEKKEEVP KASIGQLFRY TTTFDKVLLL IGSVVAIGTG
                          (1)        (2)
  61  IGLPMMSIIM GNISQNFMSI TGNTTSIQQF EHDVIQNCLK YVYLGCGVFT AATIQAMCFL

 121  TVCENLVNQL RRQFFKSILR QDITWFDKNN SGTLATKLFD NLERVKEGTG DKLGLMIQFV

 181  AQFFGGFIVA FTYDWKLTLI MMSLAPFMII CGAFIAKLMA SAATREAQKY AVAGGIAEEV

 241  LTSMRTVIAF NGQPYECERY EKALEDGKST GIKKSLYIGI GLGITFLIMF SSYCLAFWVG

 301  TDFVFKNQMQ GGTVMTVFFS VMMGSMALGQ VGPQFAVLGT AMGAAGSLYQ IIDREPEIDS

 361  YSSEGIRPSN LKGKITVSNL KFTYPTRPDV PILKGVSFEA KPGETIALVG SSGCGKSTII

 421  QLLLRYYNPA DGKITIDGVE IDKINIEFLR NYVGVVSQEP MLFNTTIEQN IRYGREKVTD

 481  AEITAALRKA NAYNFVQSFP DGIYTNVGDR GTQMSGGQKQ RIAIARALVR DPKILLLDEA

 541  TSALDAESEH IVQQALENAS KGRTTIVIAH RLSTIRNADK IIAMKNGEVV EVGNHDELIA

 601  RKGLYHELVN AQVFADVDDT VGDAAVRRRT MSSSRSRSPS LASPEYKRLR SQLSVTEDTG

 661  VATAQNDPVK AEKDLERLKK ELEEEGAAKA NLFGILRHAR PEWPFIMFAV FSSVVQGCVF

 721  PAFSLFFSQI INVFSKQPGD PTLKQEGHFW ALMFLVLGAV QATTMIIQCF FFGMSAERLT

 781  MRLRSKIFKN VMRMDATYFD MPRHSPGKIT TRLATDAPNV KSALDYRFGS VFSSVVSVCS

 841  GVGIALYFGW QMAILTIAIF PLAAVGQAIQ MKFMSGRATA DAKEMENSGK VAMEAIENIR

 901  TVQALTLEHR LHAQFCQHLD APHKTSRRKA IIQGISYGFA SSIFYFLYAS CFRFGLWLIV

 961  NGTLQPMNVL RVLFAISFTA GSMGFASSYF PEYIKATFAA GLIFHMLEEE PRIDGMTSNG
                                  (3)
1021  KKPKITGAVK LNKVYFKYPE RPDVPILQGM DVDVKPGETL ALVGPSGCGK STVISLLERL
                         (4)
1081  YDALDGSVEV DGNDLRQVNP THLRAHIALV SQEPILFDRS IRDNILYGLP PGSVSDAQVH

1141  EVAQRANIHS FIIGLPDGYN TRAGEKGAQL SGGQKQRIAI ARALVRNPKI LLLDEATSAL

1201  DTESEKVVQE ALDKASEGRT CIVVAHRLST VVNANCIMVV KGGKVVEKGT HNELMQAKGA

1261  YWALTQKQIL AKE*
```

S9 Fig. Deduced amino acid sequence of Tci-PGP-9 showing relative positions of amino acid substitutions (shaded) associated with the multiple-anthelmintic resistant RS^3 strain of *Teladorsagia circumcincta* relative to the anthelmintic susceptible S$_{\text{inbred}}$ counterpart. Alternative splicing and exon exclusion (underlined by dotted line) were detected only in RS^3 strain worms. Regions representing the putative N-terminal and C-terminal “inter-nucleotide binding domains” (IBDA and IBDB, respectively) are underlined with a solid line. Mutations described from the putative transmembrane regions amplified in the present study (see S7 Fig.**)**

resulted in three amino acid substitutions found exclusively in RS^3 strain worms which included: (1) Asn79Ser; (2) Thr86Ser; (3) Asn1043Asp. The position of an amino acid substitution resulting from a mutation in *Tci-pgp-9-IBDB* which was similarly found exclusively in RS^3 strain worms is also shown: (4) Glu1097Gln.
