# Supplementary material for: Genomic introgression mapping of field-derived multiple-anthelmintic resistance in Teladorsagia circumcincta
Source: PLoS Genet. 2017 Jun 23;13(6):e1006857. doi: 10.1371/journal.pgen.1006857 (PMC5507320; doi:10.1371/journal.pgen.1006857)
Supplement: S10 Fig — (PDF) [file pgen.1006857.s010.pdf]

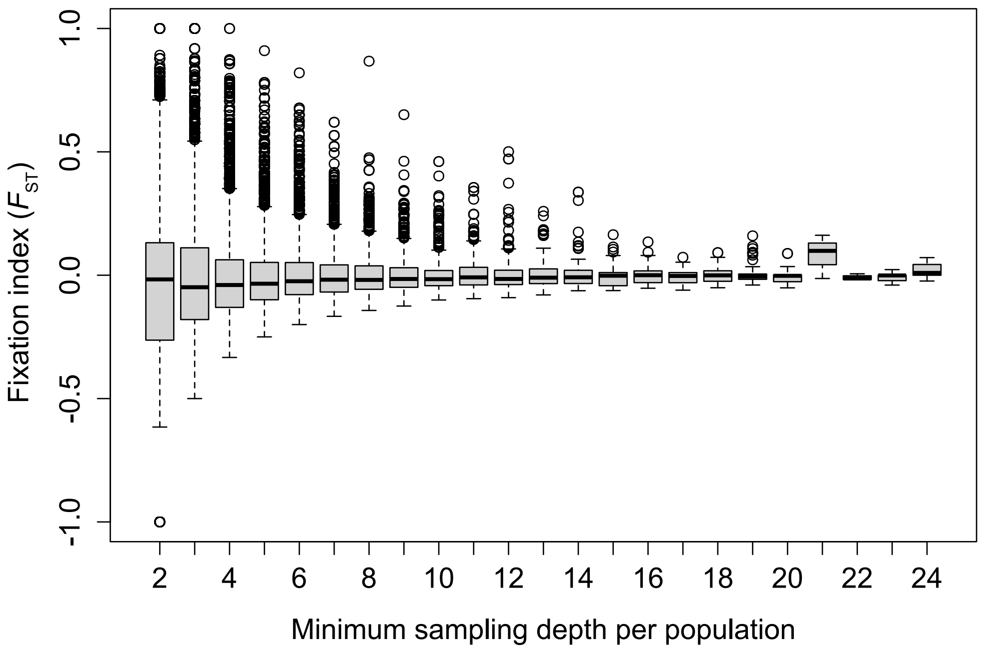

S10 Fig. Fixation index (F_{ST}) of ddRAD-seq derived SNP markers between IVM-screened and drug-naïve F2 mapping populations of *Teladorsagia circumcincta* (n = 24 male worms for each population). The upper and lower whiskers represent maximum and minimum values outside the interquartile range (middle 50%) excluding outliers (i.e., values greater than Q3 or less than Q1 by more than 1.5 times the interquartile range).
