# Supplementary material for: Genomic introgression mapping of field-derived multiple-anthelmintic resistance in Teladorsagia circumcincta
Source: PLoS Genet. 2017 Jun 23;13(6):e1006857. doi: 10.1371/journal.pgen.1006857 (PMC5507320; doi:10.1371/journal.pgen.1006857)
Supplement: S1 Table — (PDF) [file pgen.1006857.s011.pdf]

S1 Table. Summary of *Teladorsagia circumcincta* genomic features and comparison to other clade V* nematodes.

	T. circumcincta	*H. contortus*	*N. americanus*	*C. elegans*
Assembly statistics				
Total number of supercontigs (>1kb)	81730	14733	11713	7
Total length (Mb) in supercontigs	700.6	365.1	244.0	100.3
Number of N50 supercontigs	3153	1123	283	n/a
N50 supercontig length (kb)	47.1	84.7	213.1	n/a
Number of N90 supercontigs	28621	5162	1336	n/a
N90 supercontig length (kb)	2.3	13.2	29.2	n/a
GC content of whole genome	45%	43%	40%	35%
Repetitive sequences	39%	38%	24%	19%
CEGs	428 (93.4%)	431 (94.1%)	439 (95.9%)	458 (100%)
Protein-coding loci				
Total number of protein-coding genes	25532	24747	19151	20495
Avg. gene locus footprint (bp)	6415	5455	4289	3065
Avg. number of exons per gene	5.2	10	6.4	6.4
Avg. exon size (bp)	122	123	125	230
Avg. intron size (bp)	1364	445	642	339
Avg. intergenic space (bp)	11938	7821	6631	2109

* Phylogeny based on Blaxter et al. (1998)
