# Supplementary material for: Genomic introgression mapping of field-derived multiple-anthelmintic resistance in Teladorsagia circumcincta
Source: PLoS Genet. 2017 Jun 23;13(6):e1006857. doi: 10.1371/journal.pgen.1006857 (PMC5507320; doi:10.1371/journal.pgen.1006857)
Supplement: S2 Table — (PDF) [file pgen.1006857.s012.pdf]

**S2 Table. Amino acid composition (%)**

| Amino Acid | | *T. circumcincta* | *N. americanus* | *C. elegans* |
|---|---|---|---|---|
| F | Phe | 3.9 | 4.2 | 4.5 |
| S | Ser | 7.3 | 7.8 | 8.2 |
| T | Thr | 5.6 | 5.6 | 5.9 |
| N | Asn | 4.0 | 4.2 | 4.8 |
| K | Lys | 5.9 | 5.7 | 6.3 |
| E | Glu | 6.8 | 6.7 | 6.7 |
| Y | Tyr | 3.1 | 3.0 | 3.1 |
| V | Val | 6.7 | 6.7 | 6.2 |
| Q | Gln | 3.8 | 3.9 | 4.2 |
| M | Met | 2.7 | 2.6 | 2.6 |
| C | Cys | 2.1 | 2.2 | 2.0 |
| L | Leu | 8.6 | 8.9 | 8.5 |
| A | Ala | 7.4 | 7.1 | 6.5 |
| W | Typ | 1.2 | 1.1 | 1.1 |
| P | Pro | 5.3 | 5.1 | 5.1 |
| H | His | 2.4 | 2.4 | 2.3 |
| D | Asp | 5.6 | 5.4 | 5.4 |
| I | Ile | 5.2 | 5.4 | 6.0 |
| R | Arg | 6.1 | 6.2 | 5.3 |
| G | Gly | 6.2 | 5.8 | 5.4 |
