# Supplementary material for: Genomic introgression mapping of field-derived multiple-anthelmintic resistance in Teladorsagia circumcincta
Source: PLoS Genet. 2017 Jun 23;13(6):e1006857. doi: 10.1371/journal.pgen.1006857 (PMC5507320; doi:10.1371/journal.pgen.1006857)
Supplement: S4 Table — (PDF) [file pgen.1006857.s014.pdf]

S4 Table. Length of the *Teladorsagia circumcincta* genome and coding sequences (CDS) that are considered "callable" based on mapping coverage and quality.

Number of bases	All genomic regions				CDS			
	S_{inbred}		RS^3		S_{inbred}		RS^3	
Total reference	700607159		700607159		16445831		16445831	
Total Ns	124497389	(17.8%)	124497389	(17.8%)	0	(0.0%)	0	(0.0%)
No coverage	670582	(0.1%)	2265770	(0.3%)	7978	(0.0%)	13731	(0.1%)
Low coverage[a]	1473974	(0.2%)	9431101	(1.3%)	12161	(0.1%)	92656	(0.6%
Excessive coverage[b]	6735180	(1.0%)	7640561	(1.1%)	255575	(1.6%)	347097	(2.1%)
Poor mapping quality[c]	39362345	(5.6%)	68975368	(9.8%)	360944	(2.2%)	971608	(5.9%)
Callable	527867689	(75.3%)	487796970	(69.6%)	15815852	(96.2%)	15027418	(91.4%)
Callable in both strains	482349949	(68.8%)	482349949	(68.8%)	14892959	(90.6%)	14892959	(90.6%)
Callable in both strains (coverage >25x)	316492511	(45.2%)	316492511	(45.2%)	11671009	(71.0%)	11671009	(71.0%)

[a] Depth of less than 4× was considered low coverage.

[b] Depth of more than [median depth + (median absolute deviation × 1.4826) × 3] was considered excessive coverage.

[c] For loci above 10× coverage, if the fraction of reads with mapping quality < 2 exceeded 10% at a base, the site was considered poorly mapped.
