# Supplementary material for: Genomic introgression mapping of field-derived multiple-anthelmintic resistance in Teladorsagia circumcincta
Source: PLoS Genet. 2017 Jun 23;13(6):e1006857. doi: 10.1371/journal.pgen.1006857 (PMC5507320; doi:10.1371/journal.pgen.1006857)
Supplement: S5 Table — (PDF) [file pgen.1006857.s015.pdf]

S5 Table. Number of fixed and segregating bi-allelic SNPs in *Teladorsagia circumcincta* S_{inbred} and RS^3 strains. Total bi-allelic SNPs = 17,176,467. (a) Differentially fixed SNPs; (b) private SNPs in S_{inbred} population; (c) private SNPs in RS^3 population. “Reference allele” for a given SNP refers to the nucleotide base on the S_{inbred} genome assembly at the SNP's position. “Polymorphic” refers to the presence of both the reference and the alternative alleles at a given site in the respective population.

			S_{inbred}	
		Fixed<br>(reference allele)	Polymorphic	Fixed<br>(alternative allele)
		9,765,015<br>56.85%	7,354,798<br>42.82%	56,654<br>0.33%
RS^3	Fixed<br>(reference allele)			
	426,276<br>2.48%		(b)<br>422,182<br>2.46%	(a)<br>4,094<br>0.02%
RS^3	Polymorphic			
	16,489,377<br>96.00%	(c)<br>9,617,901<br>55.99%	6,851,544<br>39.89%	(c)<br>19,932<br>0.12%
RS^3	Fixed<br>(alternative allele)			
	260,814<br>1.52%	(a)<br>147,114<br>0.86%	(b)<br>81,072<br>0.47%	32,628<br>0.19%
