# Supplementary material for: Genomic introgression mapping of field-derived multiple-anthelmintic resistance in Teladorsagia circumcincta
Source: PLoS Genet. 2017 Jun 23;13(6):e1006857. doi: 10.1371/journal.pgen.1006857 (PMC5507320; doi:10.1371/journal.pgen.1006857)
Supplement: S9 Table — (PDF) [file pgen.1006857.s019.pdf]

S9 Table. Prevalence of β-tubulin genotypes in inbred anthelmintic susceptible (S_{inbred}) and multiple-anthelmintic resistant (RS^3) populations of *Teladorsagia circumcincta*.

Genotype* (aa198/aa200)	Worm population	
	S_{inbred} (n=94)	RS^3 (n=79)
$S_{198}S_{198}/S_{200}S_{200}$	94 (100.0%)	0 (0.0%)
$S_{198}S_{198}/R_{200}R_{200}$	0 (0.0%)	63 (79.7%)
$S_{198}R_{198}/S_{200}R_{200}$	0 (0.0%)	13 (16.5%)
$R_{198}R_{198}/S_{200}S_{200}$	0 (0.0%)	3 (3.8%)

* S198 – Glu in aa position 198; R198 – Leu in aa position 198; S200 – Phe in aa position 200; R200 – Tyr in aa positon 200
