# Supplementary material for: Genomic introgression mapping of field-derived multiple-anthelmintic resistance in Teladorsagia circumcincta
Source: PLoS Genet. 2017 Jun 23;13(6):e1006857. doi: 10.1371/journal.pgen.1006857 (PMC5507320; doi:10.1371/journal.pgen.1006857)
Supplement: S10 Table — (PDF) [file pgen.1006857.s020.pdf]

S10 Table. Summary of ΔC_T values in relation to the occurrence of *Tci-pgp-9-IBDA* haplotypes in male worms of the RS[3] strain of *Teladorsagia circumcincta.*

Number of worms	IBDA haplotypes present	IBDA haplotypes not present	Mean ΔCT value (range)
18	Haplotype 3		-0.09* (-1.78 – 1.03)
20	Haplotype 6	Haplotype 3	0.33* (-1.62 – 2.97)
11	Haplotype 10		-0.84* (-1.78 – 0.25)
8	Haplotype 2	Haplotypes 3, 6, 10	3.46 (2.52 – 5.08)
5	Haplotype 8	Haplotypes 3, 6, 10	3.23 (2.63 – 4.07)
5	Haplotype 9	Haplotypes 3, 6, 10	3.34 (2.52 – 5.08)

* Significantly lower than ΔC_T values from S_{inbred} worms overall (two-sample *t*-test, $P < 0.001$)
