# Supplementary material for: Genomic introgression mapping of field-derived multiple-anthelmintic resistance in Teladorsagia circumcincta
Source: PLoS Genet. 2017 Jun 23;13(6):e1006857. doi: 10.1371/journal.pgen.1006857 (PMC5507320; doi:10.1371/journal.pgen.1006857)
Supplement: S12 Table — (PDF) [file pgen.1006857.s022.pdf]

**S12 Table. Genes displaying a minimum $F_{ST}$ z-score of 2.5 in both the introgression and the F2 mapping experiments**

| Contig ID | Gene ID | | $F_{ST}$ z-score Introgression mapping | IVM F2 mapping | Minimum score |
|---|---|---|---|---|---|
| Cont209 | TELCIR_02985 | Triacylglycerol lipase | 3.4 | 4.5 | 3.4 |
| Cont209 | TELCIR_02988 | Triacylglycerol lipase | 3.3 | 4.5 | 3.3 |
| Cont228 | TELCIR_03131 | ABC transporter (*Tci-mrp-6*) | 4.2 | 3.0 | 3.0 |
| Cont228 | TELCIR_03132 | Hypothetical protein | 3.7 | 3.0 | 3.0 |
| Cont133 | TELCIR_02272 | Conserved domain protein | 2.8 | 4.0 | 2.8 |
| Cont133 | TELCIR_02271 | 6-phosphofructo-2-kinase | 2.8 | 4.0 | 2.8 |
| Cont6655 | TELCIR_15612 | Conserved domain protein | 2.8 | 4.5 | 2.8 |
| Cont6655 | TELCIR_15613 | Putative lipoyl synthase | 2.7 | 4.5 | 2.7 |
| Cont209 | TELCIR_02984 | Conserved domain protein | 2.6 | 4.5 | 2.6 |
| Cont228 | TELCIR_03125 | Hypothetical protein | 2.6 | 3.0 | 2.6 |
| Cont6655 | TELCIR_15611 | Ground-like domain protein | 2.6 | 4.5 | 2.6 |
| Cont228 | TELCIR_03126 | Hypothetical protein | 2.6 | 3.0 | 2.6 |
| Cont133 | TELCIR_02273 | Hypothetical protein | 2.5 | 4.0 | 2.5 |
| Cont5 | TELCIR_00170 | Ligand-gated anion channel (*Tci-lgc-54*) | 5.0 | 2.5 | 2.5 |
| Cont5 | TELCIR_00169 | Conserved domain protein | 4.7 | 2.5 | 2.5 |
| Cont5 | TELCIR_00168 | PHD-finger | 4.4 | 2.5 | 2.5 |
| Cont5 | TELCIR_00174 | Conserved hypothetical protein | 3.8 | 2.5 | 2.5 |
| Cont5 | TELCIR_00167 | Protein-tyrosine phosphatase | 3.7 | 2.5 | 2.5 |
