# Supplementary material for: Genomic introgression mapping of field-derived multiple-anthelmintic resistance in Teladorsagia circumcincta
Source: PLoS Genet. 2017 Jun 23;13(6):e1006857. doi: 10.1371/journal.pgen.1006857 (PMC5507320; doi:10.1371/journal.pgen.1006857)
Supplement: S14 Table — (PDF) [file pgen.1006857.s024.pdf]

S14 Table. Summary of experimental design showing lamb treatment groups, *T. circumcincta* infecting doses, final group sizes (*N*), ivermectin treatment status and 2° anthelmintic treatments applied.

Group	Infecting dose (R_{par} x S_{inbred} F_2)	*N*	Ivermectin treatment (day 27 p.i.)	Mean FEC* [range] (day 30 p.i.)	2° treatment (day 31 p.i.)
1a	8000	9	No	718 [100-2500]	Untreated
1b	8000	9	No	713 [100-1800]	Oxfendazole
1c	8000	9	No	706 [100-2000]	Levamisole
2a	16000	9	Yes	282 [0-1100]	Untreated
2b	16000	9	Yes	270 [0-1500]	Oxfendazole
2c	16000	9	Yes	315 [100-900]	Levamisole

* FEC data presented as back-transformed square-root means with range of actual counts in parentheses.
