# Supplementary material for: Genomic introgression mapping of field-derived multiple-anthelmintic resistance in Teladorsagia circumcincta
Source: PLoS Genet. 2017 Jun 23;13(6):e1006857. doi: 10.1371/journal.pgen.1006857 (PMC5507320; doi:10.1371/journal.pgen.1006857)
Supplement: S15 Table — (PDF) [file pgen.1006857.s025.pdf]

S15 Table. Between-infection-group comparisons of post-mortem worm burdens, following oxfendazole or levamisole treatment, in host animals infected with ivermectin-screened or unscreened R_{par} x S_{inbred} F_2 generation *Teladorsagia circumcincta*.

2° treatment	*N*	*T. circumcincta* infection group	Worm count relative to untreated control[a]
Oxfendazole	9	Unscreened	0.333
	9	IVM-screened	0.413[b]
Levamisole	9	Unscreened	0.305
	9	IVM-screened	0.329[b]

[a] To permit between-infection-group comparisons of post-treatment worm burdens (i.e., IVM-screened vs. unscreened populations), counts were adjusted for differences in initial worm population size by dividing them by the back-transformed square-root mean count of the untreated control group for that population. The “standardised counts” were then analysed by ANOVA after square root transformation. Pre-planned pair-wise comparisons were made using least significant differences (LSD) on the transformed scale.

[b] Within 2° treatment groups, no significant differences in “standardised worm counts” between IVM-screened and unscreened *T. circumcincta* populations. LSD ($P < 0.05$) = 0.235 for both within-treatment comparisons.
