# Supplementary material for: Genomic introgression mapping of field-derived multiple-anthelmintic resistance in Teladorsagia circumcincta
Source: PLoS Genet. 2017 Jun 23;13(6):e1006857. doi: 10.1371/journal.pgen.1006857 (PMC5507320; doi:10.1371/journal.pgen.1006857)
Supplement: S16 Table — (PDF) [file pgen.1006857.s026.pdf]

S16 Table. Summary of sequenced gDNA and mRNA libraries

Source	Platform	Strain	Library type	Mean length (bp)	Read counts	Total yield (Mb)	SRA Accession No.
gDNA	Roche/454	S_{inbred}	Fragment	377	11120366	4192	SRX499428
gDNA	Roche/454	S_{inbred}	Paired-end (3kb insert)	377	14921987	5626	SRX499427<br>SRX499429<br>SRX499430
gDNA	Roche/454	S_{inbred}	Paired-end (8kb insert)	323	3022915	976	SRX499431<br>SRX499432
gDNA	Illumina GAII	S_{inbred}	Paired-end	75	54958674	4122	SRX2011764<br>SRX2011765
gDNA	Illumina MiSeq	S_{inbred}	Paired-end	150	11811572	1772	SRX1561328
gDNA	Illumina HiSeq	S_{inbred}	Paired-end	125	474430546	59304	SRX2018770
gDNA	Illumina HiSeq	RS^3	Paired-end	100	198700496	19870	SRX1507699
gDNA	Illumina HiSeq	RS^3	Paired-end	125	368669324	46084	SRX2018769
gDNA	Illumina MiSeq	N/A	Single-end ddRAD-seq	150	26404702	3961	SRX2911816<br>SRX2911817<br>SRX2911818<br>SRX2911819<br>SRX2911820<br>SRX2911821<br>SRX2911822<br>SRX2911823<br>SRX2911824<br>SRX2911825<br>SRX2911826<br>SRX2911827
mRNA	Roche/454	S_{inbred}	Fragment	232	491725	114	SRX089125
mRNA	Roche/454	RS^3	Fragment	216	411596	89	SRX090067
mRNA	Illumina HiSeq	S_{inbred}	Paired-end	100	154058344	15406	SRX1507698
mRNA	Illumina HiSeq	RS^3	Paired-end	100	144934812	14494	SRX1507697
