# Supplementary material for: Genomic introgression mapping of field-derived multiple-anthelmintic resistance in Teladorsagia circumcincta
Source: PLoS Genet. 2017 Jun 23;13(6):e1006857. doi: 10.1371/journal.pgen.1006857 (PMC5507320; doi:10.1371/journal.pgen.1006857)
Supplement: S17 Table — (PDF) [file pgen.1006857.s027.pdf]

S17 Table. Allele-specific reverse PCR primers used in *Tci-pgp-9-IBDA* genotyping reactions

Haplotype	Primer Name	Primer Sequence	Product Size
1	IBD77RAS7	5′ GTAGATTCCCTGAAATAAGCTCAC 3′	455bp
2	IBD77RAS11	5′ CTTTTCCAGCGACGACCCGC 3′	286bp
3 (& 4)	IBD77RAS12	5′ AGCGCCATTCCACCACTTTCTTAG 3′	292bp
4	IBD77RAS3	5′ CTGTCTGAAATCTGCCTTCTCC 3′	403bp
5	IBD77RAS4	5′ CGTACTGTGGCGATCTCGA 3′	458bp
6	IBD77RAS5B	5′ AGCTGAAAGGCAGAGTCAGAG 3′	610bp
7	IBD77RAS2	5′ CGGTGTGATAAACATTGGGAGAG 3′	359bp
8	IBD77RAS8A	5′ TCCTGCCCTCTCCCTCTCAAC 3′	323bp
9	IBD77RAS9A	5′ GTGTGATAACGTCGGGGAAGATC 3′	356bp
10	IBD77RAS10C	5′ GAGTAGTCCTACAACACCGCT 3′	341bp
